# Temperature sensitive nanogel-stabilized pickering emulsion of fluoroalkane for ultrasound guiding vascular embolization therapy

**DOI:** 10.1186/s12951-023-02181-x

**Published:** 2023-11-09

**Authors:** Ling Li, Yanyan Cao, Haining Zhang, Min Zheng, Jun Xing, Chuansheng Zheng, Yanbing Zhao, Xiangliang Yang

**Affiliations:** 1 School of Biomedical Engineering and Imaging, Xianning Medical College, Hubei University of Science and Technolog, Xianning, 437100 People’s Republic of China; 2 Department of Radiology, Hubei Province Key Laboratory of Molecular Imaging, Union Hospital, Tongji Medical College, Huazhong University of Science and Technology, Wuhan, 430022 China; 3 Key Laboratory of Molecular Biophysics of Ministry of Education, College of Life Science and Technology, National Engineering Research Center for Nanomedicine, Huazhong University of Science and Technology, Wuhan City, 430074 People’s Republic of China; 4 Hubei Key Laboratory of Bioinorganic Chemistry and Materia Medical, Hubei Engineering Research Center for Biomaterials and Medical Protective Materials, Huazhong University of Science and Technology, Wuhan City, 430074 People’s Republic of China

**Keywords:** Ultrasound-guided, Fluoroalkane pickering emulsion, Ultrasound imaging, Trans-artery chemo-embolization, Temperature-sensitive

## Abstract

Various X-ray imaging technologies like computed tomography (CT) and digital subtraction angiography (DSA) are widely used in transcatheter arterial embolization (TAE) therapy for treating hepatocellular cancer (HCC) patients. Although they display high-contrast imaging, they have a few disadvantages, such as complex operation and exposure to ionizing radiation. Thus, ultrasound (US) imaging plays an important role in medical diagnosis because of its advantages, like simple and fast operation, no ionizing radiation exposure, and accurate real-time imaging. Subsequently, Poly N-isopropylacrylamide-co-2,2,3,4,4,4-Hexafluorobutyl methacrylate (PNF) nanogels were synthesized for stabilizing TGFPE, the Pickering emulsions of 2H, 3H-decafluoropentane (HDFP). These emulsions displayed dual abilities of thermosensitive sol–gel transition and long-term US imaging in vitro. Thus, it was concluded that these emulsions could achieve vascular embolization and long-term US imaging i*n vivo* as per the TAE animal model results. The emulsion droplets’ flow and accumulation were visualized under the US imaging guidance. In summary, the Pickering emulsions have the potential to be used as US-guided embolization material for mediating TAE surgeries.

**Supplementary Information:**

The online version contains supplementary material available at 10.1186/s12951-023-02181-x.

## Introduction

Due to its rising incidence rate, hepatocellular carcinoma (HCC) is the fourth leading cause of cancer-related mortality worldwide [1, 2]. Since most HCC patients present with advanced disease,  > 85% of them are not eligible for curative treatments like surgical resection, transplantation, or ablation [3]. Trans-Arterial Embolization (TAE) or Trans-Arterial Chemo-Embolization (TACE) technique selectively delivers therapeutic agents to target arteries under the guidance of medical imaging devices and is now used as a first-line treatment for advanced HCC patients [4–6]. Under the guidance of DSA, TACE selectively delivers embolization and therapeutic agents to HCC-feeding arteries through slender catheters, thereby blocking the intratumoral blood flow and releasing the drug [7, 8]. However, DSA causes ionizing radiation exposure, which is harmful to humans [9, 10]. Additionally, heavy DSA equipment is difficult to operate. Thus, ultrasound (US) is a non-invasive technique that provides real-time cross-sectional images of soft tissues and blood vessels in comparison to other imaging methods [2].

In the current clinical scenario, US-guided technology has become one of the widely used medical diagnostic procedures [11–13]. Due to poor US image resolution regarding the blood flow, Color Doppler US is used for measuring blood flow in blood vessels or deeper tissues. Thus, distinguishing normal tissues from diseased tissues becomes difficult in several cases. Hence, an ultrasonic contrast agent (UCA) is used to improve the diagnosis in such patients [14]. UCA significantly improves US image resolution and helps in the accurate detection of tiny blood vessels and tissue perfusion. Since UCA administration has several advantages like good imaging effects, real-time imaging, ease of operation, no ionizing radiation exposure, and wider application, it is increasingly used in medical diagnosis [14, 15]. Currently, microbubbles (SonoVue) are widely used as a contrast imaging medium in the diagnosis of liver cancer [16, 17]. Moreover, microbubbles suffer from inherent drawbacks, such as low stability and short half-life in the blood, due to the rapid gas diffusion through microbubble defects [18, 19]. Some liquid fluoroalkanes with boiling points between 20 and 60 °C, such as perfluorobutane (PFB), perfluorohexane (PFH), and 2H, 3H-Decafluoropentane (HDFP), are widely used for exploring newer contrast-enhancing modalities because of their high chemical inertia, hydrophobic and oilophobic properties, strong volatility and high molecular weight [20–24]. Many studies have shown that liquid fluoroalkane Pickering emulsions have higher stability, longer in vivo cycle times, and better-targeted tissue accumulation. Concurrently, they can also be vaporized under ultrasonic stimulation to produce improved US images when compared to SonoVue.

Pickering emulsion is an emulsion stabilized by inorganic or polymeric nanoparticles like silica, gold, and polystyrene nanoparticles. They have garnered more attention than classical surfactant-stabilized emulsions due to their advantages, like high physical/chemical stabilities, excellent biocompatibility, and environmental affinity [25–27]. Nanogels have been extensively employed as drug nanocarriers due to their high stability, excellent drug loading capability, tunable surface property, and responsiveness to various microenvironmental stimuli [28, 29]. Therefore, temperature-sensitive poly (N-isopropylacrylamide) (*p*NIPAM) nanogels have been successfully used as emulsifiers for Pickering emulsions, as they demonstrated sustained drug release, prolonged in vivo blood circulation and enhanced antitumor efficacy [30]. However, at an increased temperature above their low critical solution temperature (LCST, ca. 32 °C), the *p*NIPAM-nanogel-stabilized Pickering emulsion might become unstable and undergo oil–water phase separation. This inherently limits the applicability of *p*NIPAM-nanogel-stabilized Pickering emulsion as sustained/controlled release carriers and biomedical materials. Furthermore, random copolymerization of monomers, such as methacrylic acid (MAA), resulted in *p*(NIPAM-*co*-MAA) nanogels that could be used to stabilize octanol-in-water emulsion at 60 °C and pH of 9.4. Because of their hydrophilic nature, the nanogels were significantly influenced by temperature due to the enhanced MAA ionization. Moreover, neutral pH caused the instability of the emulsion stabilized by *p*(NIPAM-*co*-MAA) nanogels, thereby resulting in droplet coarsening and demulsification [31, 32]. Hence, improving the stability of *p*NIPAM nanogel-stabilizing Pickering emulsion above LCST and retaining its temperature sensitivity is crucial for its biomedical application.

**Scheme 1:**
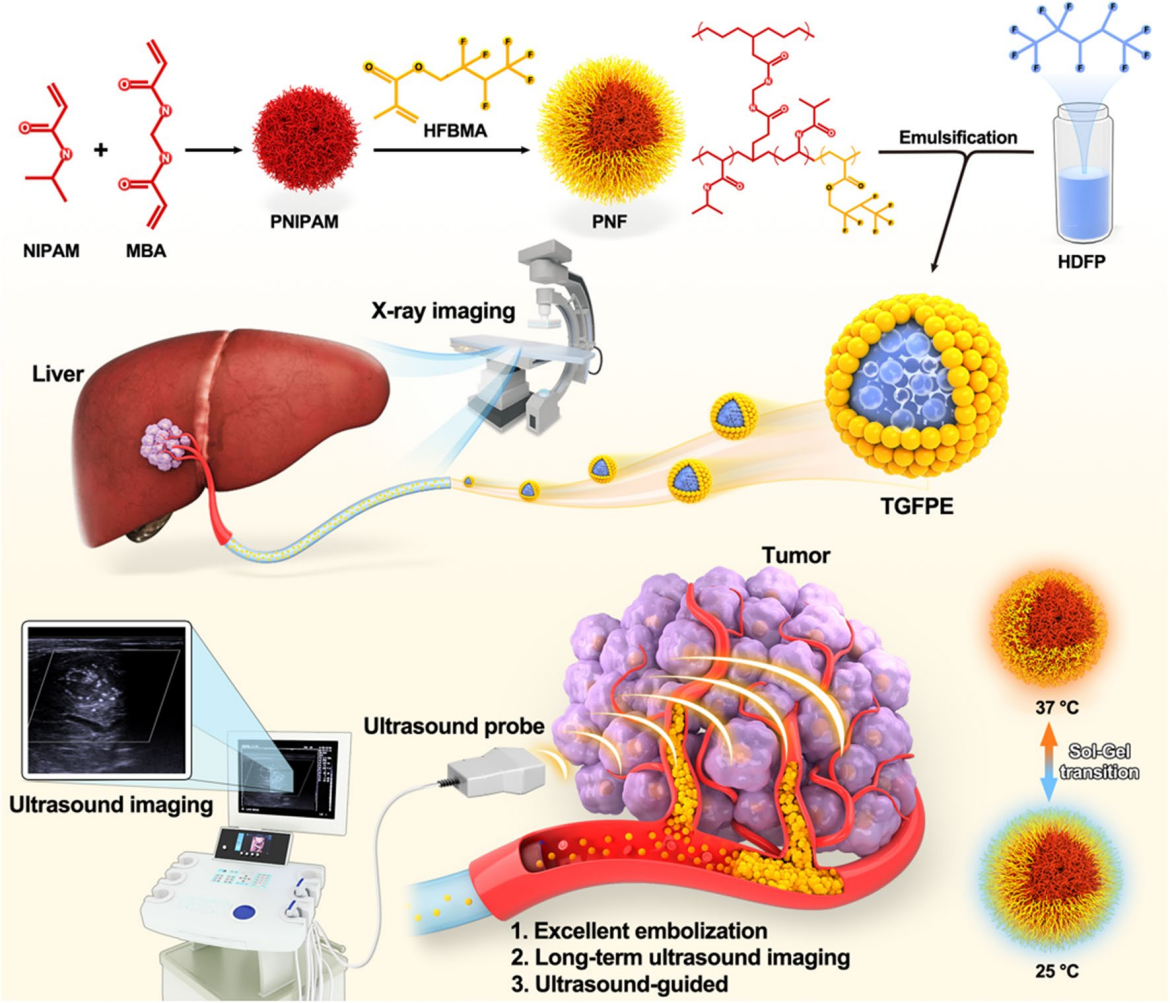
Schemematic illustration of Pickering emulsion of 2H, 3H-decafluoropentane (HDFP) stabilized by thermosensitive fluorine-containing nanogel (TGFPE) for enhancing antitumor efficacy of blood-vessel embolization

Thus, we designed the Pickering emulsion of HDFP, stabilized by thermosensitive fluorine-containing nanogel (TGFPE). Through catheter-directed movement, TGFPE could target the tumor vasculature and transit from sol state to gel state under 37 °C, thereby asserting its role as an embolic material. After gel formation, the emulsion droplets were enmeshed into the gel network to prolong their time in tumor vessels and to achieve long-term US imaging in tumors. Furthermore, the TGFPE emulsion’s flow and accumulation could be visualized under the US imaging guidance in vivo. Hence, this could be evidence to assert that US imaging technology can be used for guiding TAE and TGFPE therapies in clinical settings (Scheme 1).

## Results and discussion

### The synthesis and characterization of Poly (*N*-isopropylacrylamide-*co*-2,2,3,4,4,4-Hexafluorobutyl methacrylate) (PNF) nanogels

**Fig. 1:**
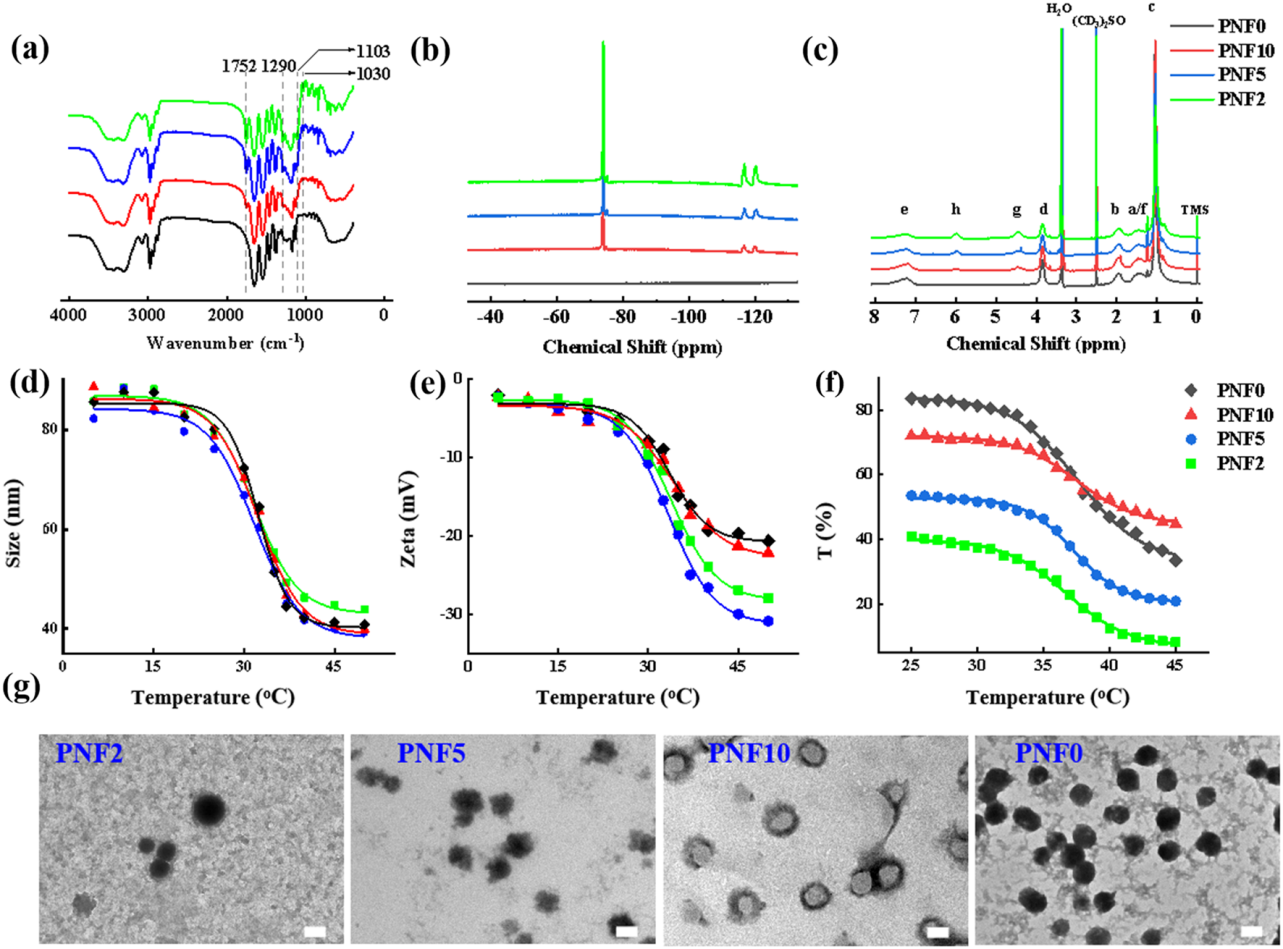
Characterizations of PNF nanogels. **a** Fourier transform infrared (FTIR) spectra, **b** ^9^F-NMR spectrums, **c** ^1^H-NMR spectrums of different PNF nanogels. **d** size, **e** Zeta potential and **f** transmittance of different PNF nanogels dispersion. **g** TEM characterization of PNF nanogels, Scale = 50 nm

*p*NIPAM nanogels were mainly polymerized using 2,2,3,4,4,4-Hexafluorobutyl methacrylate (HFBMA) to improve their affinity toward fluoroalkanes owing to their hydrophobic and lipophobic properties. Moreover, PNF nanogels were synthesized by the two-step seed emulsion polymerization method (Additional file 1: Fig. S1) as per the synthesis formula shown in Additional file 1: Table S1. Moreover, the PNF nanogel structure could be altered by adjusting the addition order of reaction monomers. However, *p*NIPAM nanogels, the core of PNF nanogels, were initially synthesized by the first-step emulsion polymerization. Consequently, NIPAM conversions of approximately 95% were observed after a reaction for 30 min (Additional file 1: Fig. S2). Consequently, HFBMA was added to form the shell of linear *p*HFBMA. The PNF nanogel structures were regulated by the feeding ratio of monomers (NIPAM/HFBMA) and were subsequently named PNF2 (2/1), PNF5 (5/1), PNF10 (10/1), and PNF0 (no HFBMA) according to the feeding ratio, respectively. The chemical structure of PNF nanogels was characterized by NMR and FT-IR measurements. The absorption peaks of C = O (1752 cm^–1^) in -COOC- groups, C-F (1290 cm^–1^, 1103 cm^–1^, and 1030 cm^–1^) were observed in the FT-IR spectra of PNF10, PNF5, and PNF2 nanogels; however, enhanced absorption peaks were perceived with increased HFBMA usage (Fig. 1a). In the ^9^F-NMR and ^1^H-NMR spectra of PNF10, PNF5 and PNF2, the fluorine and hydrogen signal peaks (the peaks at a chemical shift of 4.5 and 6.0 ppm belong to -O-CH_2_- and -CHF-, respectively) were observed in the linear PHFBMA segment (Fig. 1b and c). The above results indicated that the HFBMA monomers were polymerized successfully on the *p*NIPAM surface. Furthermore, the molecular ratios of NIPAM and HFBMA and the HFBMA conversion rate were calculated conforming to the ^1^H-NMR results through formulation 1. The molecular ratios of PNF10, PNF5, and PNF2 were 16.7, 7.1, and 2.9, while their conversion rates were 59.88%, 70.42%, and 68.97%, respectively. Thus, our findings were similar to the elemental analysis results (Additional file 1: Table S2).

The hydrodynamic diameter, Zeta potential, and light transmittance of PNF nanogel dispersions were measured to detect their temperature-sensitive property (Fig. 1d–f). However, the variation trends of PNF nanogels’ size were similar with rising temperatures. Although hydrodynamic diameters of PNF nanogels were approximately 80 nm at 25 °C, they reduced to 45 nm at temperatures > 37 °C. The VPTTs of PNF2 (31.61 °C), PNF5 (31.65 °C), PNF10 (32.25 °C), and PNF0 (32.35 °C) diminished, thereby suggesting that the PNF nanogels’ VPTT decreased as HFBMA percentage increased (Fig. 1d). Furthermore, elevated temperature led to an increase in the specific surface area of PNF due to the nanogel’s contraction. Consequently, the PNF nanogel’s Zeta potential value increased and was considered stable. Notably, the absolute values of the Zeta potential of PNF nanogels increased as the HFBMA content escalated owing to the strong electronegativity of the F atom (Fig. 1e). The LCSTs of PNF2 (36.48 °C), PNF5 (37.28 °C), PNF10 (37.49 °C) and PNF0 (37.59 °C) were acquired after analyzing the light transmittance data. Thus, the variation trend of LCST was similar to the abovementioned result (Fig. 1f). It might be because the longer the linear PHFBMA, the more hydrophobic PNF nanogels were. However, the hydrophobic PHFBMA did not affect the VPTT and LCST of core–shell PNF nanogel dispersion. This finding was contrary to our earlier conclusion that the hydrophobic units had a big influence on VPTT and LCST of random-homopolymer nanogels [33]. The TEM imaging displayed the core–shell structures of PNF10, PNF5, and PNF2 (Fig. 1g). The above results showed successful synthesis of core–shell fluorinated nanogels. Otherwise, PNF nanogels had excellent temperature-sensitive properties.

A contrast agent, iohexol (240 mg I/mL), was administered before x-ray imaging to reveal the passage of the material into blood vessels during subsequent animal experiments. The NaCl concentration can affect the polymer’s surface charge and its sol–gel phase transition behavior in aqueous dispersions. Thus, NaCl was added to adjust the gelling behavior of PNF nanogel dispersion, while iohexol was administered to aid in the injection of embolic materials under the guidance of DSA imaging. Our results revealed that the dispersion with 0.5 wt.% NaCl could gel well at 37 °C temperature (Additional file 1: Fig. S3).

### Screening the formulation of TGFPE

**Fig. 2:**
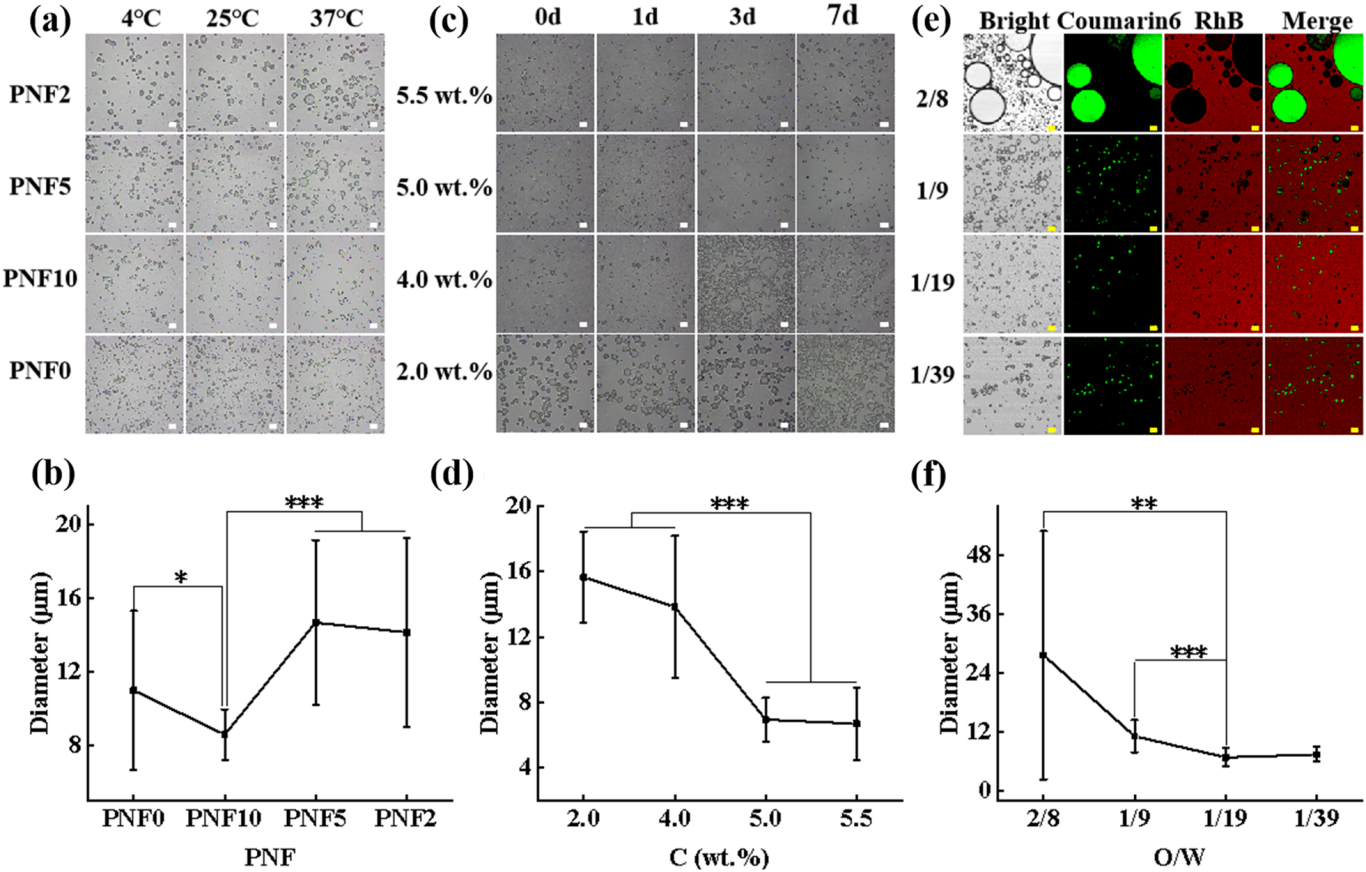
The screening of TGFPE fomulation. **a** The optical microscopic photos of TGFPE stabilized by different PNF10 nanogels after treating at different temperature. **b** The statistical analysis of droplets’ size in TGFPE. **c** The optical microscopic of TGFPE stabilized by PNF10 nanogel dispersion with different concentrasion at different points of time. **d** The statistical analysis of droplets’ size in TGFPE. **e** The fluorescent images of fluorescence labelled TGFPE with different ratios of aqueous phase (5.5 wt.% PNF-RhB-10 dispersion) and oil phase (HDFP labelled by Coumarin 6). **f** The statistical analysis of droplets’ size in TGFPE. Scale = 20 μm, n = 20 for (**a**), (**b**) and (**c**)

The effect of PNF nanogel composition on the stability of TGFPE emulsion was investigated by stabilizing the TGFPE emulsion by PNF2, PNF5, PNF10, and PNF0 nanogels, respectively. The TGFPE emulsion changes after constant temperature treatment at 4 °C, 25 °C, and 37 °C were observed by optical microscope. It was observed that the size of emulsion droplets did not significantly differ under room temperature, but the droplets enlarged and their sizes increased because of the coalescence after 37 °C treatment except for the TGFPE group that was stabilized by PNF10 (Fig. 2a). A quantitative analysis revealed that the mean size of TGFPE droplets stabilized by different PNF nanogels after treatment at 37 °C were 14.15 ± 5.15 µm (PNF2), 16.69 ± 4.48 µm (PNF5), 8.58 ± 1.38 µm (PNF10) and 10.99 ± 4.32 µm (PNF0), respectively (Fig. 2b). Since TGFPE stabilized by PNF10 nanogels had smaller size and narrow size droplets, the PNF10 was considered for stabilizing the last emulsion.

We further examined the influence of PNF10 concentration on the stability of TGFPE emulsions. After initial preparation, the TGFPE droplets stabilized by 2 wt.% PNF10 nanogel dispersion sank to the bottom of the bottle, and their sizes were the biggest, thereby suggesting that the emulsion stabilized by 2 wt.% PNF10 nanogel dispersion had the worst stability. Moreover, the TGFPE droplets stabilized by 4 wt.% and 5.0 wt.% PNF10 nanogel dispersion sank due to coalescence after preparing for 2 and 5 days, respectively. However, the size of droplets in the former condition enlarged due to coalescence, but the droplets in the latter did not differ significantly. Only the TGFPE droplets stabilized by 5.5 wt.% PNF10 nanogel dispersion did not sink and enlarged simultaneously after a week’s preparation (Fig. 2c and Additional file 1: Fig. S4). A quantitative analysis suggested that the mean sizes of TGFPEs stabilized by PNF10 dispersions with different concentrations were 14.84 ± 2.72 µm, 13.03 ± 4.36 µm, 6.95 ± 1.33 µm, and 6.66 ± 2.38 µm, respectively (Fig. 2d). To sum up, the stability of TGFPE emulsions improved along with increased concentration of PNF10 nanogel dispersion. Nonetheless, the TGFPE emulsions did not easily deform if they were stabilized by higher concentrations of PNF10 nanogel dispersion, thus limiting their applicability (Additional file 1: Fig. S5).

Besides the above two aspects, the ratio of oil and aqueous phases (O/W) is one of the most important factors that affect the stability of emulsions. From the fluorescence-labeled TGFPE images, the sizes and distribution of droplets in TGFPE emulsions with different O/W were compared (Fig. 2e and f). When the O/W was 2/8, the size of TGFPE droplets was 27.63 ± 25.33 µm, suggesting that a stable emulsion could not be achieved under this condition. With increasing O/W of TGFPE, the size of TGFPE droplets was calculated as 11.14 ± 3.32 µm of 1/9, 6.81 ± 1.88 µm of 1/19 and 7.39 ± 1.49 µm of 1/39, respectively. Moreover, the size distributions of TGFPE droplets were uniform as O/W was 1/9, 1/19, and 1/39, respectively. The size of TGFPE droplets having O/W of 1/9 was bigger than the other two emulsions. Through the fluorescence distribution region, we observed that the oil phase was surrounded by the water phase as a disperse phase, thereby suggesting that our emulsions were of O/W type.

We also perceived that the intensity of fluorescence around the droplets emitted from rhodamine B (RhB) was stronger than other areas of TGFPE (Fig. 2e). This result suggested that PNF10 nanogels were clustered at the two-phase interface, indicating that they successfully functioned as stabilizers. Furthermore, we observed the TGFPE structure under TEM and noticed that there was a circle with a dark color, thus proving our earlier conclusion (Additional file 1: Fig. S6).

### Phase transition behavior and ultrasonic development performance of TGFPE in vitro

**Fig. 3:**
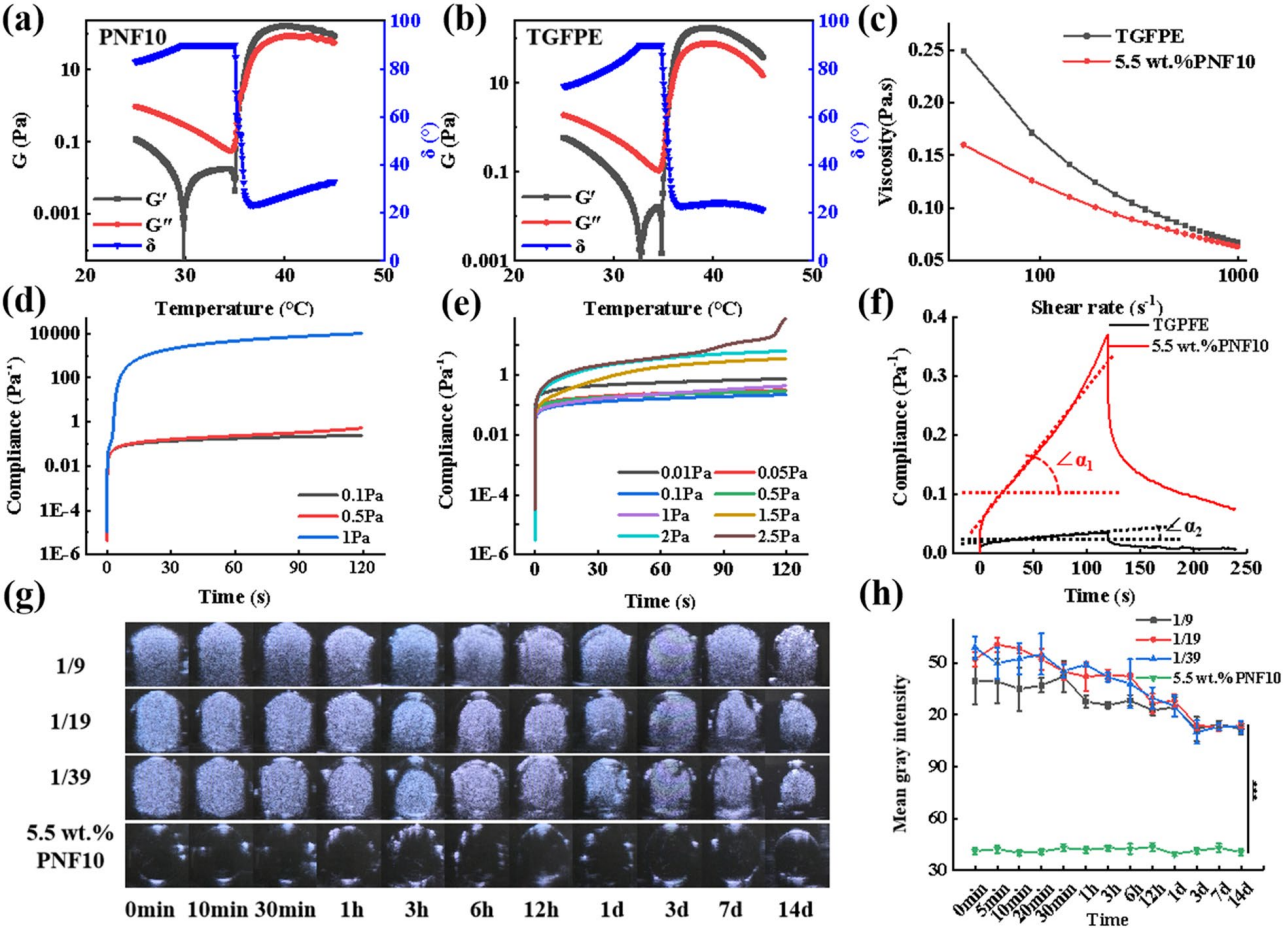
Characterizations of rheological and ultrasound imaging of TGFPE. The shear modulus at different temperature of **a** PNF10 nanogel dispersion and **b** TGFPE. **c** The comparation of shear viscosity at different shear rate of PNF10 nanogel dispersion and TGFPE. Yield stress detection of **d** PNF10 nanogel dispersion and **e** TGFPE. **f** Creep and creep recovery testing of PNF10 nanogel dispersion and TGFPE. **g** Ultrasound imaging performances of TGFPE with different ratios of aqueous phase and oil phase and PNF10 nanogel dispersion. **h** The statistical analysis of mean gray intensity (n = 5)

Rheological properties can be used to characterize the ability of embolic materials for vascular embolization. In order to monitor the sol–gel state, the modulus of TGFPE emulsions and PNF10 nanogel dispersions, comprising storage and loss modulus, was measured under different temperatures from 25 to 45 °C. As the temperature rose from 25 to 35 °C, their modulus decreased due to enhanced flowability. As temperature further rose to 37 °C, their modulus increased rapidly. As their storage modulus was bigger than the loss modulus, they transited to a gel state, and the corresponding temperature was named sol–gel phase transition temperature (Fig. 3a and b). Additionally, the TGFPE emulsions, at different concentrations after gelling under 37 °C, resulted in an enhanced TGFPE complex modulus as the concentration of PNF10 nanogel dispersions increased. This suggested that the gelation strength of TGFPE could be strengthened by increasing the PNF10 nanogel dispersion concentration. Moreover, the TGFPE emulsion at an O/W ratio of 1/19 had a higher complex modulus than other TGFPE emulsions with other O/W ratios (Additional file 1: Fig. S7a). An assessment of the viscosity of TGFPE emulsions under the same conditions revealed that the viscosity of TGFPE emulsions rose as the concentration of PNF10 nanogel dispersions increased. Thus, the stability of TGFPE emulsions could be improved if they were stabilized by PNF10 nanogel dispersions with higher concentrations. The TGFPE having an O/W of 1/19 had a higher modulus than others with different O/W and PNF10 nanogel dispersions, suggesting its higher stability and that the TGFPE emulsion viscosity rose as the ratio of O/W increased (Additional file 1: Fig. S7b–d). It was worth noting that TGFPE had a higher complex modulus (124.8 Pa) than PNF10 dispersion (71.8 Pa) after gelling at 37 °C, suggesting that it had better efficacy in blocking the bloodstream. We measured the TGFPE and PNF10 nanogel dispersion viscosities under different shear rates to detect their flowability, as the viscosity of embolic materials greatly influenced the TAE procedure. A reduction of 3.68 folds was noticed when the TGFPE viscosity decreased from 249.3 MPa.s under the shear rate of 41.27 s^–1^ to 67.7 MPa.s under the shear rate of 991.7 s^–1^. Concurrently, the viscosity of PNF10 nanogel dispersion decreased from 160 MPa.s under a shear rate of 41.27 s^–1^ to 63.5 MPa.s under a shear rate of 991.7 s^–1^ (Fig. 3c). Thus, it was suggested that their flowability decreased as the shear rates rose, suggesting that both of them possessed the shear thinning property. Because of this property, the resistance was reduced when they were pushed in vessels through the catheter, although they had high viscosity when stored. After gelling at 37 °C, we found a rapid increase in compliance occurring at 1 Pa and 2.5 Pa for PNF nanogel dispersions and TGFPE through the creep compliance tests, respectively (Fig. 3d and e). Thus, the result that TGFPE resisted higher shear stress post-gelling than PNF10 dispersion confirmed that TGFPE had a stronger ability to resist blood flow scouring. Furthermore, a higher TGFPE zero shear viscosity (6535.09 Pa.s) than PNF10 nanogel dispersion (398.41 Pa.s), indicated its higher mechanical strength after gelling (Fig. 3f). In conclusion, TGFPE has the potential of application in vascular embolization.

The property of US imaging of TGFPE was measured to verify whether it could be used as a US contrast agent. Thus, TGFPE had excellent US imaging performance when compared with PNF10 nanogel dispersions. Within 2 weeks after gelling at 37 °C, we found that TGFPE groups could detect significant US imaging signals within 2 weeks post-gelling and their intensity was approximately ≥ 3 times than PNF nanogel dispersion (Fig. 3g and h). According to the above results, the TGFPE with an O/W of 1/19 and stabilized by 5.5 wt.% PNF10 nanogel dispersion was chosen for later experiments. The X-ray attenuation of TGFPE was measured by CT scan, and the CT value was determined. When the proportion of Omnipaque increased from 80 to 320 mg I/mL, the CT values of TGFPE increased from 1810.31 to 3066.5. This showed that TGFPE exhibited the excellent ability of X-ray attenuation (Additional file 1: Fig. S8).

### In vivo renal artery embolization and ultrasound imaging in normal rabbits

**Fig. 4:**
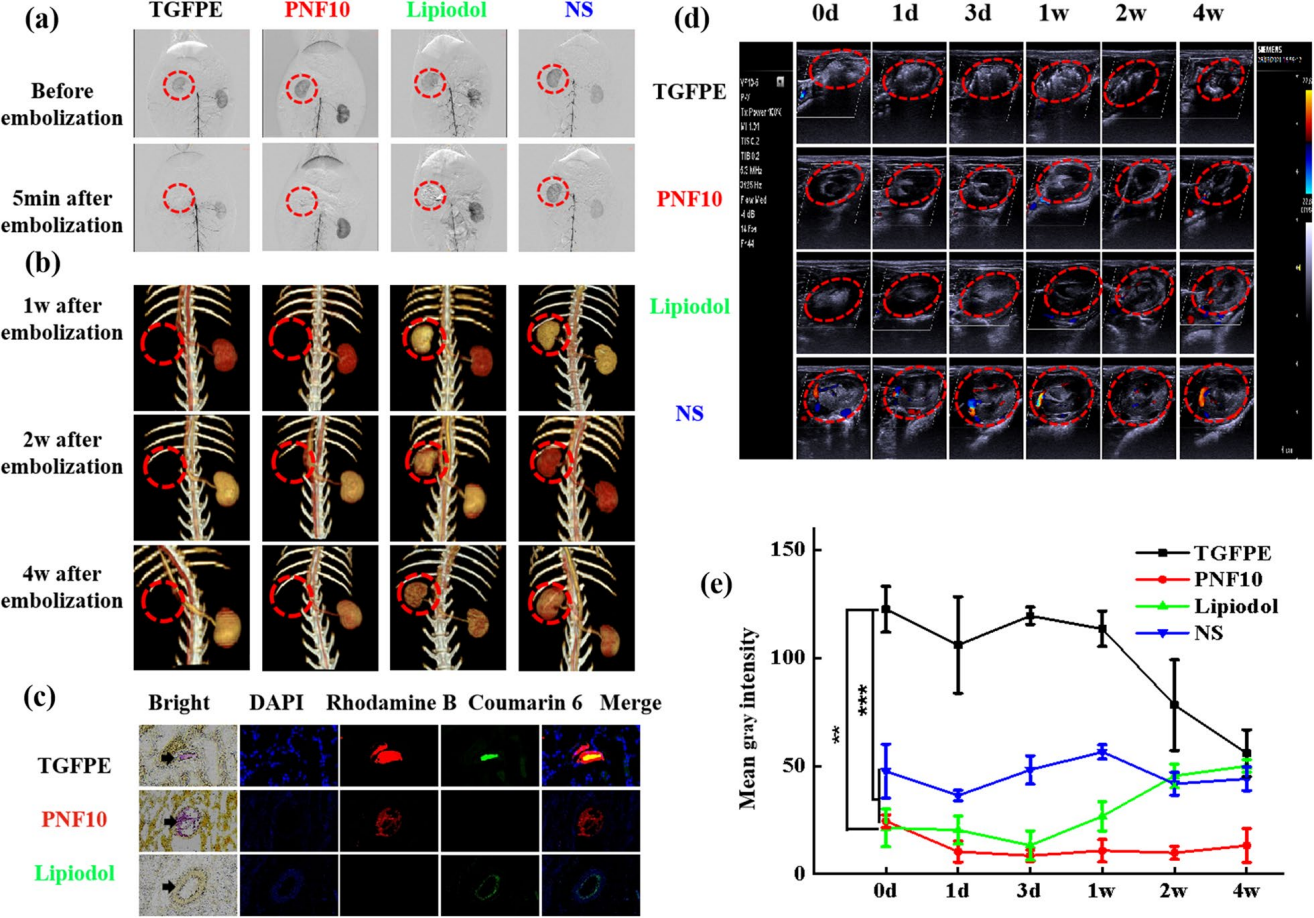
Renal artery embolization and ultrasound imaging comparison of TGFPE, PNF10 dispersion, lipiodol and NS. **a** DSA images of kidney at different points of time after vascular embolization with the TGFPE, PNF10 dispersion, lipiodol and NS. **b** 3D-CT images of kidney at different points of time after vascular embolization with the TGFPE, PNF10 dispersion, lipiodol and NS. **c** Bright field and fluorescence scanning of isolated embolized kidney slices within 1 d after vascular embolization (arrow pointing to blood vessel). **d** Color doppler and B-mode ultrasound images of kidney at different points of time after being embolized by the above embolic material or the injection of NS. **e** The statistical analysis of B-mode ultrasound images in kidneys at different points of time after being embolized by the above embolic material or the injection of NS. The region surrounded by dotted red line is the embolized kidney. **P < 0.01, ***P < 0.001

Renal artery embolization is usually done to evaluate the ability of embolic materials for vascular embolization and US imaging performance preliminarily [34–36]. Under DSA imaging, a catheter was introduced near the entrance of the main renal artery. After the passage of iohexol into the kidney, the TGFPE and PNF10 nanogel dispersions were injected into the kidney vasculature. The kidney was not visible in DSA imaging after the injection of TGFPE and PNF10 nanogel dispersion for 5 min, suggesting the formation of a good vascular embolization (Fig. 4a). Enhanced CT examination was further conducted to evaluate the revascularization of the kidney. As shown in Fig. 4b, abundant blood signals were detected in the kidneys of the NS group. The signals weakened after vascular embolization by lipiodol due to its embolism properties, but they returned to normal levels later owing to vascular recanalization. Within 4 weeks after vascular embolization, blood flow signals were not observed in the kidney of TGFPE and PNF10 groups, suggesting that the vasculature was still blocked by the gel networks formed by TGFPE or PNF nanogel dispersion. One day after vascular embolization, kidneys embolized by fluorescently-labeled embolic materials were isolated and their slices were observed by fluorescence imaging. Moreover, we found that the TGFPE emulsion and PNF10 nanogel dispersion remained intact in the vessel, while the fluorescence signal emitted from lipiodol was only observed on the blood vessel walls (Fig. 4c). The kidneys were isolated from the body after vascular embolization for 4 weeks. Microscopically, the kidneys in the TGFPE and PNF groups shrank severely and were necrosed, but the kidneys in the lipiodol group shrank mildly without any necrosis (Additional file 1: Fig. S9a). However, H&E staining revealed an absence of normal structure of renal slices in the TGFPE and PNF10 groups, while the kidney in the lipiodol group displayed a normal structure (Additional file 1: Fig. S9b).

Enhanced Color Doppler and B-mode US echo signals were detected in the TGFPE group kidney within 4 weeks after vascular embolization, while US echo signals were weaker in the kidneys of other groups. Four weeks post-vascular embolization, the echo intensity of the TGFPE group was similar to the NS and lipidol groups but still higher than the PNF10 group (Fig. 4d and Additional file 1: Fig. S10). The mean gray value of the TGFPE group’s blood vessels was the highest within 4 weeks by the statistical analysis of B-mode US images in kidneys at different intervals after being embolized by the TGFPE, PNF10 dispersion, lipiodol, and NS (Fig. 4e). The reason for this might be that TGFPE and PNF nanogel dispersion formed a gel network for blocking the kidney’s blood supply permanently, while lipiodol embolized the vasculature temporarily. Furthermore, we located the TGFPE-embolized kidneys and determined whether the embolic materials were still inside the vessel at the embolization site by detecting significantly enhanced TGFPE signals from the vasculature. Additionally, with color doppler US, we observed whether the vascular supply was recanalized or not. Given the above findings, TGFPE could be further applied in vascular embolization as a self-US imaging embolic material.

Additionally, we also injected TGFPE and NS into the rabbit’s kidney under the guidance of US imaging. Firstly, NS was injected into the kidney’s vascular supply. However, we could not observe the process of injecting these materials. After injecting TGFPE, an increased US imaging intensity was observed in the kidney (Additional file 1: Fig. S11 and Additional file 2: Video S1, Additional file 3: Video S2). The flow and accumulation of TGFPE were monitored under US imaging (Additional file 3: Video S2). Hence, it was confirmed that US imaging could be used for precisely guiding the TAE therapy, and TGFPE could be used as a special embolic material for successful TAE therapy.

### In vivo evaluation of TAE and imaging efficacy in VX2 tumor-bearing rabbits

**Fig. 5:**
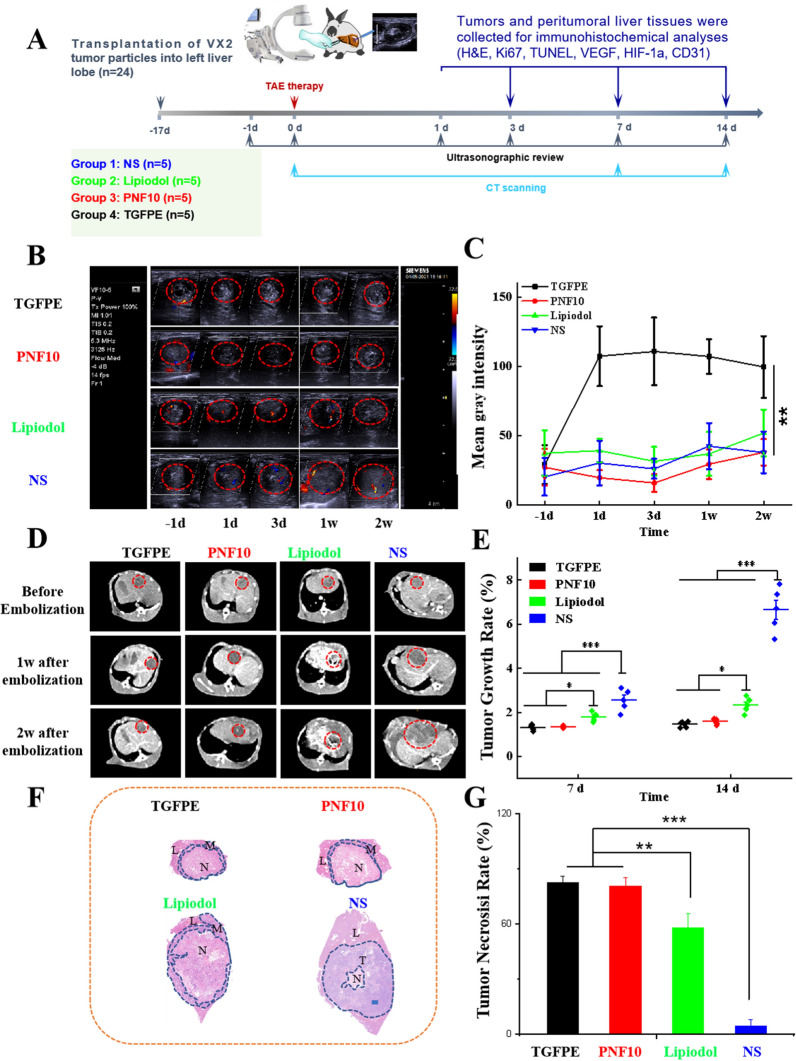
Antitumor efficacies of TAE therapy and ultrasound imaging comparison of TGFPE, PNF10 dispersion, lipiodol and NS in VX2 tumor-bearing rabbits. **a** Schematic illustration of experiment schedule. **b** Color doppler and B-mode ultrasonography images in tumors before and at different points of time after being embolized by the above embolic material. The region surrounded by dotted red line is the embolized kidney. **c** The statistical analysis of B-mode ultrasound images. **d** CT images of tumors before and at different points of time after being embolized by the above embolic material. The region surrounded by dotted red line is the embolized tumor. **e** The statistical analysis of tumor sizes at 1 day before and after vascular embolization with the above embolic materials or the injection of NS. **f** The H&E stainning of tumor after vascular embolization with the above embolic materials for 2 weeks. (T) areas of tumor (L) liver tissue (N) areas of necrosis (M) tumor margins. **g** The statistical analysis of tumor necrosis rates after vascular embolization with the above embolic materials for 2 weeks. Scale = 2500 μm. n = 5, *P < 0.05, **P < 0.01, ***P < 0.001

In order to evaluate the antitumor efficacy of embolization (Fig. 5a), 24 VX2-tumor-bearing rabbits were randomly divided into four groups: NS, lipiodol, PNF10, and TGFPE. Compared with a normal liver area, the tumor area was covered by a chaotic vascular network under DSA imaging. Based on this, tumors could be found easily, and we could inject embolic materials into them precisely. After TGFPE, PNF10 nanogel dispersion and lipiodol were injected into the tumor vasculature for 5 min. However, the vascular network that was observed before its disappearance owing to the X-ray contrast agent could not flow into it (Additional file 1: Fig. S12), thus suggesting that the tumor vasculature was adequately embolized. Color Doppler and B-mode US were used to evaluate the tumor revascularization (Fig. 5b and c). Moreover, several bloodstream signals were observed near the tumor in the control group, NS, during the whole experimental period. The bloodstream signals at the tumor weakened after embolization by lipiodol, but the signals persisted. Compared with the above-mentioned results, the blood flow signals were absent in the tumor’s vascular network within 2 weeks after vascular embolization in color doppler US imaging. This suggested that the gel networks formed by TGFPE and PNF10 nanogel dispersion were not still washed by the bloodstream and remained in vascular supply. Enhanced US-imaging intensity from tumors in the TGFPE group was detected within 2 weeks after vascular embolization under B-mode US, while in other groups, weakened echo signals were observed in the tumor areas during the whole experimental period. The trend of tumor growth was monitored by US imaging (Fig. 5b and Additional file 1: Fig. S13). We also found that the tumor in the NS group enlarged significantly as time went by, showing a trend of tumor growth that was not inhibited completely. Although the growth of tumors was inhibited due to the embolization effect of lipiodol, their sizes still increased significantly 2 weeks after TAE therapy. However, no significant tumor enlargement could be found in the TGFPE and PNF 10 groups. In order to measure the size of tumors accurately, the tumor growth trend was monitored by CT imaging (Fig. 5c). Compared with the NS group, which showed a suppressed tumor growth trend, the growth of tumors in the TGFPE, PNF10, and lipiodol groups were all suppressed, after a 2-week TAE therapy. Moreover, the tumor growth rates in the TGFPE, PNF10, lipiodol, and NS groups were 1.46 ± 0.14, 1.60 ± 0.13, 2.33 ± 0.26, and 6.65 ± 1.00 respectively, thereby suggesting that TGFPE and PNF10 nanogel dispersion displayed higher efficacy in suppressing tumor growth than lipiodol after gelling in vivo (Fig. 5d). After a 2-week TAE treatment, tumors showed coagulative necrosis except for tumors in the NS group while the tumors in the TGFPE and PNF10 groups showed complete necrosis (Fig. 5f and Additional file 1: Figs. S14, S15). Compared with them, the lipiodol group tumors showed some active areas, suggesting that lipiodol could not block the blood supply of tumors completely and that the lipiodol-induced vascular embolization recanalized easily. For measuring the tumor necrosis rates of all groups, tumor slices were stained by H&E and scanned microscopically. An intergroup comparison revealed that the TGFPE (81.96% ± 3.48%) and PNF10 (80.96% ± 4.66%) groups had higher tumor necrosis rates than lipiodol (61.44% ± 8.37%) and NS (5.21% ± 3.33%) groups (Fig. 5g). To sum up, TGFPE displayed good efficacy for intratumoral vascular embolization and long-term US imaging guidance.

### The evaluation of post-operative tumor microenvironment

**Fig. 6:**
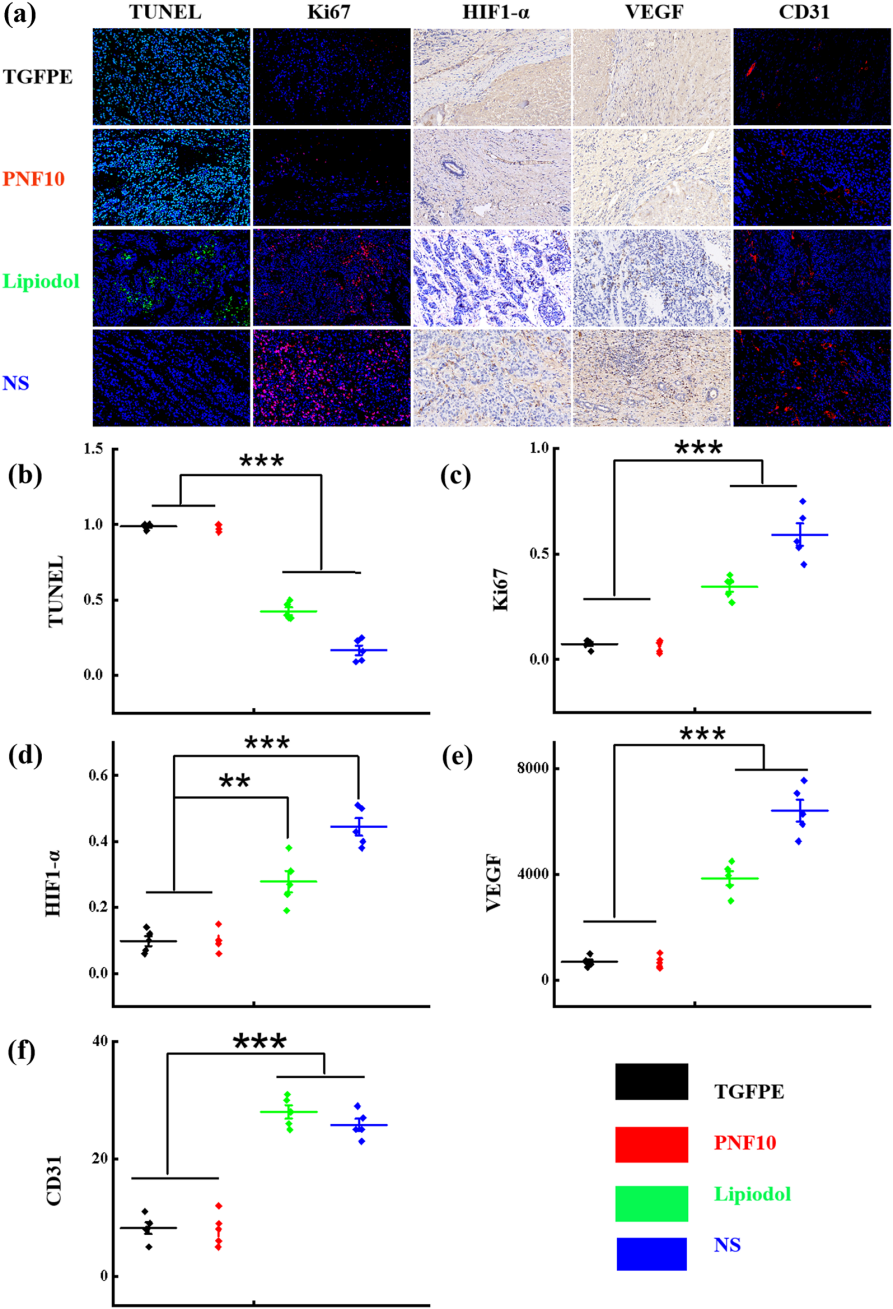
Immunohistochemical evaluations on the neovascularization of VX2 tumor-bearing rabbits with various treatments on day 14 (NS, lipiodol, PNF10, TGFPE). **a** Confocal fluorescence microscopy images of TUNEL staining, Ki67 staining, HIF-1α staining, VEGF staining and CD31 staining in the residual tumor surrounding the tumor necrosis zone (original magnification, × 400). **b**, **c**, **d**, **e**, and **f** are a quantitative comparison of fluorescence intensities in the slices of TUNEL staining, Ki67 staining, HIF-1α staining, VEGF staining, and CD31 staining from plot A, respectively. **P < 0.01, ***P < 0.001

Embolization denotes the interruption of the blood flow to the tumors, thereby inducing ischaemic necrosis and tumor hypoxia. Although hypoxia is toxic to cancer cells, its precise effects vary with its extent and duration. Severe and prolonged hypoxia induces cell death, whereas mild or short-term hypoxia can cause several adaptive genetic cellular alterations [34, 37]. The H&E-stained section revealed an extensive necrotic area, tumor structural disorganization, and a few surviving tumor cells in the VX2 tumor treated with PNF10 and TGFPE. However, many surviving tumor nidi were found in tumors treated with NS and lipiodol (Additional file 1: Fig. S15). Moreover, TUNEL staining was performed on tumor tissue sections, where an increase in the green fluorescence indicated enhanced tumor cell death in the treatment groups. An increased level of green fluorescence of TUNEL-positive nuclei was observed only in PNF10 and TGFPE-treated tissue sections, thus providing evidence of apoptotic cell death (Fig. 6a and b, and Additional file 1: Fig. S16). Additionally, reduced tumor cell proliferation was evident from the decreased Ki67 expression in the PNF10 and TGFPE treatment groups when compared with NS and lipiodol groups (Fig. 6a–c, and Additional file 1: Fig. S17). The significant differences in tumor apoptosis and proliferation indicated that PNF10 and TGFPE achieved full embolization of all levels of tumor arteries when compared with lipiodol and NS, thereby exhibiting a stronger antitumor effect.

The local hypoxia induced by insufficient TAE therapy can cause activation of HIF-1α and enhancement of the VEGF expression. Consequently, positive HIF-1α staining was noted in both the cytoplasm and nuclei of viable tumor cells. Moreover, HIF-1α-positive tumor cells were located predominantly in the peripheral necrotic tumor regions [38–40]. Compared to those with both NS and lipiodol therapies, HIF-1α, VEGF, and CD31 levels in the rabbits distinctly decreased at 14 d with the treatments of PNF10 and TGFPE.

As shown in (Fig. 6a and c), HIF-1α expressions at 14 d in the PNF10 and TGFPE-treated groups were much lower than the groups treated with NS and lipiodol; thus, demonstrating significant differences in HIF-1α expressions among the four groups. Cytoplasmic VEGF expression was detected in the viable tumor cells at the periphery of necrotic tumor regions. However, the number of VEGF-positive cells significantly increased in the NS and lipiodol groups when compared with the PNF10 and TGFPE-treated group (Fig. 6a and d). Since microvessels were heterogeneously distributed in the tumors, the most intense vascularization was observed in the invading tumor margins. Additionally, CD31 expression significantly increased on day 14 in the NS and lipiodol groups, whereas the CD31 expressions of PNF10 and TGFPE-treated groups significantly decreased (Fig. 6a–e and Additional file 1: Fig. S18). In VX2 tumors, HIF-1α, as well as CD31 expressions, showed a significant positive correlation with VEGF-positive cells. The purpose of embolization is to interrupt the tumor blood supply, thereby inducing ischaemic necrosis and tumor hypoxia. Although hypoxia is cytotoxic, the precise effect varies with its extent and duration. Moreover, severe and prolonged hypoxia induces cell death, whereas mild or short-term hypoxia causes various adaptive genetic changes in cells. Our results regarding the HIF-1α, VEGF, and CD31 expressions indicated that PNF10 and TGFPE-treated groups effectively limited neovascularization and collateral circulation when compared to NS and lipiodol groups.

### The evaluation of TGFPE’s biocompatibility

**Fig. 7:**
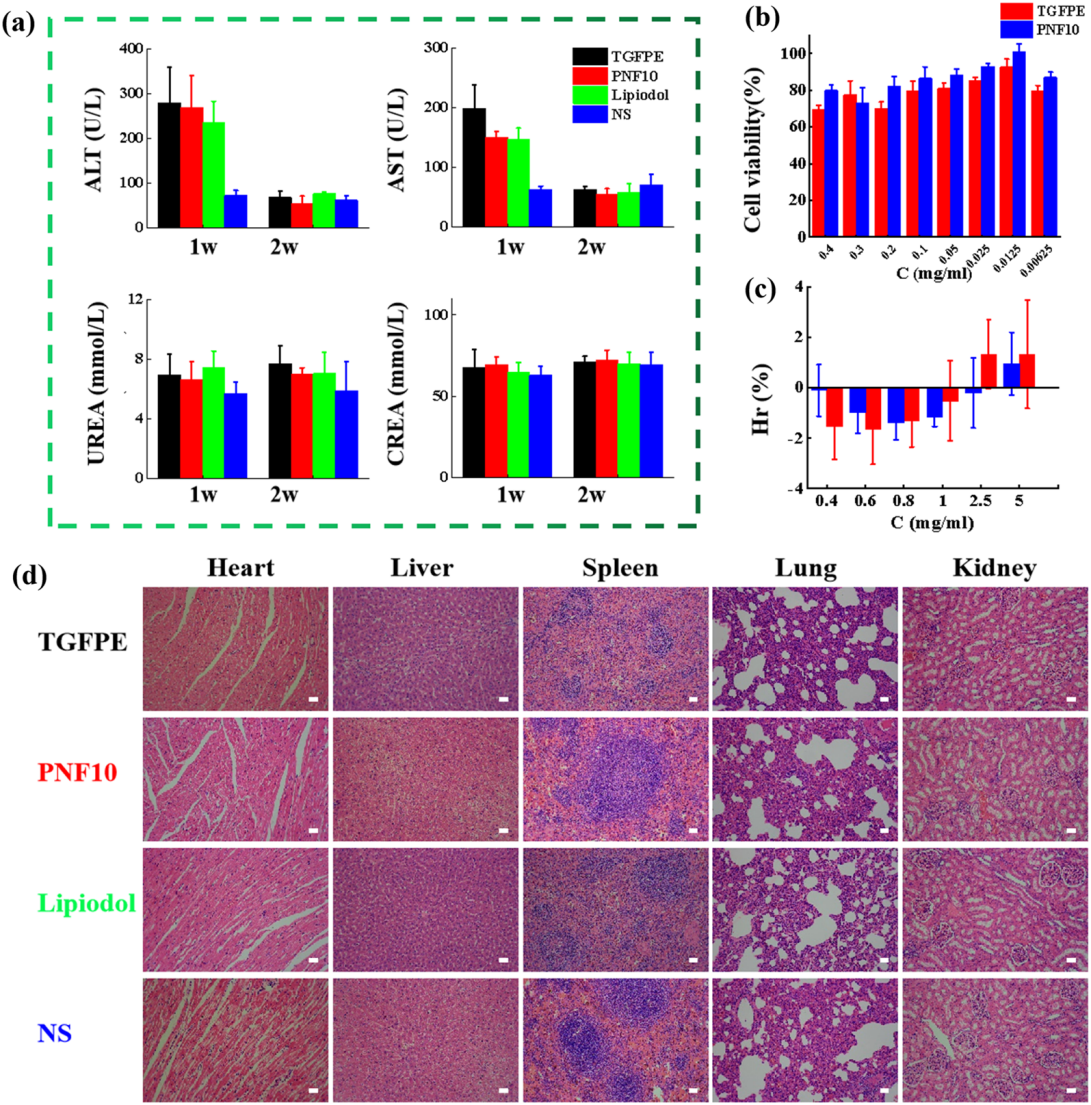
Biocompatibility evaluation. **a** Hepatorenal function of VX2 tumor-bearing rabbits for 1 weeks and 2 weeks after treatments: ALT (alanine aminotransferase), AST (aspartate aminotransferase), BUN (blood urea nitrogen), and CRE (creatinine), (n = 5). **b** Cytotoxicity comparison using the CCK-8 method on HUVEC cells (n = 6). **c** Hemolysis comparison (n = 10) of TGFPE, PNF10. **d** H&E staining of the heart, liver, spleen, lung and kidney tissue sections of the experimental rabbits in each embolization group (Scale = 20 μm)

The biocompatibility of TGFPE was evaluated from several parameters like body tissues, hepatorenal function, hemolysis ratios (Hrs), and cytotoxicity. In our study, all serums were used to detect the indexes of hepatorenal function consisting of ALT, AST, UREA, and CREA. Although the ALT and AST levels in the TGFPE, PNF10, and lipiodol groups were higher than the NS group after TAE therapy for 1 week, all indexes returned to normal after a 2-week TAE therapy, suggesting that the hepatorenal function of rabbits could not be influenced through TAE therapy by using TGFPE (Fig. 7a). The cell viability of TGFPE was > 70% when the concentration range of 0.00625–0.4 mg/mL was incubated with HUVEC cells for 24 h, indicating the low toxicity of TIPE (Fig. 7b). As shown in Fig. 7c, when the dilution concentration of the TGFPE was 0.4–5 mg/mL, the hemolysis rate was < 5% which suggested good blood compatibility. After H&E staining was done on normal organ slices from all the groups, no obvious tissue damage and lesions were observed in the slices, suggesting that there were no negative impacts on normal organs after administering TAE therapy on rabbits by using TGFPE, PNF10 dispersion, and lipiodol (Fig. 7d). Thus, we concluded that TGFPE had good biocompatibility.

## Conclusion

X-ray imaging is usually used in guiding TAE therapy and postoperative assessment, but it possesses various disadvantages like the risk of radiation, complex operation, etc. However, for using US imaging for guiding TAE therapy, which has advantages like the absence of radiation exposure and simple operation, the core–shell PNF nanogels with different molecular compositions were synthesized utilizing two-step seed emulsion polymerization. Furthermore, temperature-sensitive Pickering emulsions of HDFP and TGFPE were prepared by applying PNF nanogels as stabilizers. In these Pickering emulsions, TGFPE stabilized by PNF10 nanogels showed excellent stability that was not significantly influenced by temperature, i.e., the size and distribution of HDFP droplets were unchanged after treatment at 37 °C. Moreover, PNF10-stabilized TGFPE had a smaller and more uniform size distribution of droplets, showing higher stability (up to 1 week) at 5.5 wt.% and 5% of PNF10 nanogel concentration and oil content, respectively. The TGFPE modulus rapidly increased, and G′ became higher than G″ as temperature rose to approximately 35 °C, indicating that the TGFPE transited from sol to gel state. However, TGFPE showed excellent flowability before gelling and good gelation strength after gelling. Compared with PNF10 nanogel dispersion, it had higher yield stress and zero shear viscosity at 37 °C, indicating that TGFPE might achieve better vascular embolization. After TGFPE was used for rabbit renal artery embolization, it showed excellent long-term vascular embolization and US imaging in the kidney > 4 weeks. The evaluation of TAE antitumor efficacy and intratumor US imaging on VX2-tumor-bearing rabbits suggested that TGFPE significantly inhibited tumor growth and was apparent in long-term intratumor US imaging for up to 2 weeks. Under the US imaging guidance, the dynamics flow and accumulation of TGFPE in the kidney could be monitored, suggesting that US imaging could be further developed for guiding the TAE therapy, and TGFPE could be a crucial component involved in this process. Combined with its good biocompatibility, TGFPE has shown promising results as a novel self-imaging embolic material for TAE therapy, thereby providing better intraoperative guidance of embolic material and postoperative review.

### Supplementary Information

**Additional file 1. Supporting Figures and Tables: Table S1.** Synthesis formula of PNF nanogels. **Table S2.** The elemental analysis of PNF nanogels. **Table S3.** The structural composition of PNF nanogels. **Figure S1.** The two-step feed emulsion polymerization of PNF nanogels. **Figure S2.** Polymerization kinetics of PNIPAM core. **Figure S3.** NaCl concentration-temperature phase diagram of 5.5 wt.% PNF10 nanogel dispersion. **Figure S4.** Macroscopical observation of TGFPE emulsions stabilized by PNF10 nanogel dispersions with different concentration. **Figure S5.** Microscopic observation of TGFPE Emulsion stabilized by 6 wt.% and 7 wt.% PNF10 nanogel. Scale = 250 μm. **Figure S6. **TEM observation of TGFPE emulsion. Scale = 250 μm.** Figure S7.** Modulus and viscosity detection of TGFPE emulsions. **a)** and **b)** are modulus and viscosity detection of TGFPE emulsions stabilized by PNF10 nanogel dispersions with different concentration, respectively. **c)** and **d)** modulus and viscosity detection of TGFPE emulsions with different ratios of O/W. **Figure S8. **CT value of TGFPE at virous iodine content. (0 mg I/mL, 80 mg I/mL, 160 mg I/mL, 240 mg I/mL, and 320 mg I/mL). **Figure S9. **Histopathological evaluation of rabbit kidney embolism with of TGFPE, PNF10 dispersion, lipiodol and NS. **a)** Photos of kidneys after vascular embolization with the above embolic materials for 4 weeks and **b)** the microscopic observation of kidney slices after H&E staining. Scale = 20 μm. **Figure S10.** B-mode ultrasound images of kidney after being embolized. **Figure S11. **The observation of the whole processes of injecting NS and TGFPE into the normal kidney under the guidance of ultrasound. The region surrounded by dotted red line is the renal aorta. **Figure S12.** DSA images of tumors before and after embolized for 5 minutes. The region surrounded by dotted red line is the embolized tumor. **Figure S13.** B-mode ultrasound images of tumors after being embolized. **Figure S14.** The photos of liver and tumor after 14 days of embolization. The region where red arrow pointed at was tumor. **Figure S15.** H&E staining of different groups after embolization on day 14 (original magnification, × 100 ). (T) areas of tumor (L) liver tissue (N) areas of necrosis. **Figure S16.** Confocal fluorescence microscopy images of DAPI (blue color) and TUNEL (green color), (original magnification, ×400). **Figure S17. **Confocal fluorescence microscopy images of DAPI (blue color) and Ki67 (red color), (original magnification, ×400). **Figure S18. **Confocal fluorescence microscopy images of CD31 surrounding the residual tumors at different time points, (original magnification, ×400).

**Additional file 2.** NS was monitored under US imaging.

**Additional file 3.** TGFPE was monitored under US imaging.
