# Supplementary material for: Temperature sensitive nanogel-stabilized pickering emulsion of fluoroalkane for ultrasound guiding vascular embolization therapy
Source: J Nanobiotechnology. 2023 Nov 9;21:413. doi: 10.1186/s12951-023-02181-x (PMC10634024; doi:10.1186/s12951-023-02181-x)
Supplement: Supplementary file 1 — Additional file 1. Supporting Figures and Tables: Table S1. Synthesis formula of PNF nanogels. Table S2. The elemental analysis of PNF nanogels. Table S3. The structural composition of PNF nanogels. Figure S1. The two-step feed emulsion polymerization of PNF nanogels. Figure S2. Polymerization kinetics of PNIPAM core. Figure S3. NaCl concentration-temperature phase diagram of 5.5 wt.% PNF10 nanogel dispersion. Figure S4. Macroscopical observation of TGFPE emulsions stabilized by PNF10 nanogel dispersions with different concentration. Figure S5. Microscopic observation of TGFPE Emulsion stabilized by 6 wt.% and 7 wt.% PNF10 nanogel. Scale = 250 μm. Figure S6. TEM observation of TGFPE emulsion. Scale = 250 μm. Figure S7. Modulus and viscosity detection of TGFPE emulsions. a) and b) are modulus and viscosity detection of TGFPE emulsions stabilized by PNF10 nanogel dispersions with different concentration, respectively. c) and d) modulus and viscosity detection of TGFPE emulsions with different ratios of O/W. Figure S8. CT value of TGFPE at virous iodine content. (0 mg I/mL, 80 mg I/mL, 160 mg I/mL, 240 mg I/mL, and 320 mg I/mL). Figure S9. Histopathological evaluation of rabbit kidney embolism with of TGFPE, PNF10 dispersion, lipiodol and NS. a) Photos of kidneys after vascular embolization with the above embolic materials for 4 weeks and b) the microscopic observation of kidney slices after H&E staining. Scale = 20 μm. Figure S10. B-mode ultrasound images of kidney after being embolized. Figure S11. The observation of the whole processes of injecting NS and TGFPE into the normal kidney under the guidance of ultrasound. The region surrounded by dotted red line is the renal aorta. Figure S12. DSA images of tumors before and after embolized for 5 minutes. The region surrounded by dotted red line is the embolized tumor. Figure S13. B-mode ultrasound images of tumors after being embolized. Figure S14. The photos of liver and tumor after 14 days of embolization. The region [file 12951_2023_2181_MOESM1_ESM.pdf]

Supporting information

Temperature sensitive nanogel-stabilized pickering emulsion of fluoroalkane for ultrasound guiding vascular embolization therapy

Ling Li[1,§], Yanyan Cao[2,§], Haining Zhang[3,§], Min Zheng[1], Jun Xing[1], Chuansheng Zheng[2,*], Yanbing Zhao[3,4,*] , Xiangliang Yang[3,4*]

[1] School of Biomedical Engineering and Imaging, Xianning Medical College, Hubei University of Science and Technolog, Xianning 437100, PR China

[2] Department of Radiology, Union Hospital, Tongji Medical College, Huazhong University of Science and Technology, Hubei Province Key Laboratory of Molecular Imaging, Wuhan 430022, China.

[3] National Engineering Research Center for Nanomedicine, Key Laboratory of Molecular Biophysics of Ministry of Education, College of Life Science and Technology, Huazhong University of Science and Technology, 430074, Wuhan City, P. R. China.

[4] Hubei Key Laboratory of Bioinorganic Chemistry and Materia Medical, Hubei Engineering Research Center for Biomaterials and Medical Protective Materials, Huazhong University of Science and Technology, 430074, Wuhan City, P. R. China.

* Corresponding Authors. E-mail addresses: zhaoyb@hust.edu.cn (Y. Zhao); yangxl@mail.hust.edu.cn (X. Yang); hqzcsxh@sina.com (C. Zheng).

§ These authors contributed equally to this work.

Experimental Methods

Materials

N-isopropylacrylamide (NIPAM, J&K Scientific Co., Ltd.) and N, N'-methylene bis-acrylamide (MBA, Aladdin Bio-chemical Technology Co., Ltd.) are recrystallization with n-hexane and methanol, respectively. Sodium dodecyl sulfate (SDS, Aladdin bio-chemical technology Co., Ltd.), Potassium persulfate (KPS, Sinopharm Chemical Reagent Co., Ltd.), Sodium chloride (NaCl, Sinopharm Chemical Reagent Co., Ltd.), 2,2,3,4,4,4-Hexafluorobutyl methacrylate (HFBMA, J&K Scientific Co., Ltd.), 2-Aminoethyl methacrylate hydrochloride (AM, J&K Scientific Co., Ltd.), Rhodamine isothiocyanate B (RhB, Seebio (Shanghai) Co., Ltd.), Phosphotungstic acid (J&K Scientific Co., Ltd.), 2H,3H-Decafluoropentane (HDFP, TCI (Shanghai) Chemical Industry Development Co., Ltd.) and Coumarin 6 (TCI (Shanghai) Chemical Industry Development Co., Ltd.) are used directly without further purification. SDS-PAGE Gel Quick Preparation Kit (Beyotime Biotechnology Co., Ltd.), Hydrated chloral (Wuhan Union Hospital), Iodophor (Wuhan Union Hospital), Iohexol (Jiangsu Hengrui Pharmaceutical Co., Ltd.), Lipiodol (Guerbet France), Paraformaldehyde (Biosharp Co., Ltd.), Heparin sodium (Wuhan Union Hospital), Normal saline (NS, Henan Kelun Pharmaceutical Co., Ltd.), Hematoxylin (Beijing OKA Biotechnology Co., Ltd.), Eosin (Sigma-Aldrich), RPMI medium (Hyclone), Phosphate buffer solution (Wuhan Service biotechnology Co., Ltd.), Penicillin/streptomycin（P/S）(Gibco), Trypsin (Biosharp), Fetal Bovine Serum（FBS）(Gibco), MTT (J&K Scientific Co., Ltd.).

HUVEC cell was preserved in our lab. New zealand rabbits (2.5±0.5 kg weight, both sexes) were provided by Laboratory Animal Research Center, Tongji Medical Institute of Huazhong University of Science and Technology. All rabbits were housed in a room with controlled temperature for at least one week prior to study. All procedures were in accordance with China National Animal Law on the use of laboratory animals. All animal studies were performed according to the guidelines approved by the Institutional Animal Care and Use Committee at Tongji Medical College, Huazhong University of Science and Technology (HUST, Wuhan, China).

Synthesis of PNF nanogels

The first step emulsion polymerization for *p*NIPAM core. NIPAM (3.39 g, 30 mmol), MBA (0.231 g, 1.5 mmol), SDS (0.192 g, 6.7 mol) and 340 mL ultra-pure water were added into a 500 mL three-way flask with magnetic stirrer. 15.4 mg/mL KPS solution was prepared for later use. Reaction temperature was controlled at 70°C by oil bath. And argon gas was continuously pumped into the flask to drain the air in the reaction solution, so that the reaction was carried out under the protection of argon gas. After argon was pumped for 30 min, 7.8 mL of the KPS solution was added into the flask to initiate the first emulsion polymerization reaction, and *p*NIPAM was obtained after 30 min of polymerization reaction.

Step 2 emulsion polymerization for PNF nanogels. According to Table S1, a certain amount of HFBMA was added into the above reaction system and the reaction was continued for 4 h. At the end of the reaction, unreacted monomers and SDS were removed by dialysis in ultra-pure water for 3 days. After freeze-drying under vacuum

condition, dried thermosensitive polymers PNF2, PNF5, PNF10 and PNF0 nanogels were obtained and stored.

AM (13 mg, 0.1 mmol) and RhB (20 mg, 0.05 mmol) were added into the reaction system before the polymerization. After the reaction, The PFNF-RHB-10 nano-gel labeled with the fluorescent dye RhB was obtained.

Characterization of PNF nanogels

NIPAM monomer conversion rate of emulsion polymerization in the first step

Standard curve: Accurately weighing 4 mg NIPAM, dissolved in 10 mL ultra-pure water as mother liquor; 1 mL the above solution was accurately diluted to 10 mL, and 0.04 mg/mL NIPAM solution was obtained. Take 5 mL of the diluent of the previous step and dilute it to 10 mL in turn for getting configure 0.02 mg/mL, 0.01 mg/mL, 0.005 mg/mL, 0.0025 mg/mL, and 0.00125 mg/mL NIPAM solutions. The absorbances of standard sample solutions were measured at 220 nm wavelength. The standard curve of NIPAM concentration-absorbance was obtained by curve fitting:

$$C(mg/mL) = i + j \times Abs \qquad \text{Formulation 1}$$

Where i and j were respectively the intercept and slope of the fitted curve equation after curve fitting.

Sample detection in the experimental group: During the synthesis of PNF0 nanogels, 2 mL reaction solution was taken from the flask at 0 min, 1 min, 3 min, 5 min, 10 min, 15 min, 20 min, 25 min, 30 min, 40 min, 50 min and 60 min after the polymerization, respectively. Appropriate amount of NaCl was added to precipitate the nanogel in the reaction solution at each time point. After centrifugation at 5000

rpm for 5 min, the supernatant of the reaction solution was taken and diluted to appropriate multiples. After that, the absorbance value of the supernatant diluted at 220 nm was detected. The monomer conversion rate was calculated according to the following formula:

$$x(mg/mL) = t(i + j \times Abs) \qquad \text{Formulation 2}$$

$$\text{Conversion Rate}(\%) = (x - x_0)/x_0 \times 100 \qquad \text{Formulation 3}$$

Where x is the concentration of NIPAM in the reaction solution at each time point, x_0 is the concentration of NIPAM in the reaction solution at the reaction time point 0, and t is the dilution ratio of the supernatant.

Structure and composition characterization of PNF nanogels

Weighing 5 mg PNF nanogels, FT-IR was used to analyze the differences of characteristic absorption peaks representing different chemical bonds between different compositions of PNF nanogels. 10 mg PNF nanogels were dissolved in 500 μL DMSO-D6 and transferred into a nuclear magnetic tube for 9F-NMR and 1H-NMR detection. The ratio of [NIPAM] in the structure of PNF nanogels and the conversion rate of HFBMA monomer were calculated according to the following formula:

$$[\text{NIPAM}]:[\text{HFBMA}] = \left(\frac{S_d}{S_h}\right):1 \qquad \text{Formulation 4}$$

$$\text{Conversion Rate}(\%) = ([\text{NIPAM}]_0 \times S_h)/([\text{HFBMA}]_0 \times S_d) \qquad \text{Formulation 5}$$

Where S_d and S_h are the characteristic peak areas of PNIPAM and PHFBMA, respectively, and $[[\text{NIPAM}]_0$ and $[\text{HFBMA}]_0$ are the feed quantities of NIPAM and HFBMA respectively. 10 mg PNF nanogels were weighed and characterized by

elemental analysis. The ratio of NIPAM and HFBMA in the structure of PNF nanogels and the conversion rate of HFBMA monomer were calculated by the following formula:

$$[\mathrm{NIPAM}]:[\mathrm{HFBMA}] = \left\{\frac{8}{\left[\left(\frac{7C_C}{6C_N}\right)-6\right]}\right\}:1 \qquad \text{Formulation 6}$$

$$\text{Conversion Rate}(\%) = \frac{[\mathrm{NIPAM}]_0\times(7C_C-36C_N)}{[\mathrm{HFBMA}]_0\times 48C_N}\times 100 \qquad \text{Formulation 7}$$

Where C_C and C_N are the content of C and N in PNF nanogels , and [$[NIPAM]_0$ and $[HFBMA]_0$ are the feed quantities of NIPAM and HFBMA, respectively.

TEM characterization of PNF nanogels

10 mg dried PNF nanogels were weighed and separated in 10 mL ultra-pure water for use; 10 mg phosphotungstic acid was weighed and dissolved in 5 mL ultra-pure water for later use. 10 μL PNF nanogels dispersion was dropped onto the copper net, and blot the liquid on the copper wire with filter paper after standing for 10 min. Then adding 10 μL phosphotungstic acid solution to the copper net for 10 min. Removing the liquid from the copper net with filter paper. Place the copper net on a filter paper to dry overnight. The morphology of PNF nanogels adsorbed on the copper net was observed by transmission electron microscope (TEM; Tecnai G2 20, FEI Corp., Eindhoven, the Netherlands). TEM voltage is 200 kV.

Temperature sensibility of PNF nanogels

Size and Zeta potential characterization of PNF nanogels: 2 mL of PNF nanogels were taken after the polymerization. 1 mL sample solution was injected into the particle size cell and potential cell, and the size and zeta potential of PNF nanogels were measured at different temperature by dynamic light scattering (Zetasizer Nano

ZS90, Malvern Instruments Ltd., Malvern, UK) with a He-Ne laser source (λ = 633 nm, scattering angle: 90°) .

LCST detection of PNF nanogels: 50 mg PNF nanogels were Weighed and seperated it in 5 mL ultra-pure water for later use. 2 mL of PNF nanogel dispersions were added into a quartz colorimetric dish and the transmittance of them at 500 nm wavelength was detected at different temperatures (25-45°C, temperature interval was 1°C).

Effect of NaCl concentration on gelation behavior of PNF nanogels *in vitro*: 5.5 wt.% PNF10 nanogel dispersion was prepared with 240 mg I/mL iohexol solution and different NaCl concentrations as aqueous phase. The samples were placed in water bath for 5 min to observe their states at different temperatures.

Preparation of TGFPE emulsion

PNF nanogel dispersions with different concentrations of PNF nanogel dispersions were obtained by evenly dispersing in 240 mgI/mL iohexol solution with 0.5 wt.% NaCl after agitation overnight. The aqueous dispersions were mix with HDFP according to different oil-water ratios, and the system was subjected to high-shear emulsification at 9000 RPM under ice water bath conditions. Emulsification time was 120 s (shear 30 s, stand 30 s and repeat 4 times) to obtain TGFPE emulsion with different components.

Characterization of TGFPE emulsion

Screening of TGFPE emulsion formula

PNF nanogels with different compositions: TGFPE emulsions stabilized by different PNF nanogels were placed at 4°C, 25°C and 37°C for 10 min, respectively. After the sample temperature returned to room temperature, the morphology of the sample droplets in TGFPE emulsions were observed by optical microscope.

Different concentrations of PNF10: At different time points, the droplets morphology and size of TGFPE emulsion stabilized with different concentrations of PNF10 were observed under optical microscope. Macroscopic sedimentation phenomenon of TGFPE emulsions were observed by naked eye.

Different O/W: Coumarin-labeled HDFP and RHB-labeled PFNF-RHB-10 nanogel were used to prepare corresponding TGFPE emulsion. The micro-morphology of TGFPE emulsion droplets with different O/W was observed by laser scanning confocal microscope under excitation light of 488 nm and 546 nm.

TEM characterization of TGFPE emulsion

Dilute the original liquid of TGFPE emulsion twice. Referring to the operation of TEM characterization of PNF nanogels, the microscopic morphology of droplets in TGFPE emulsion was observed by TEM.

Rheological characterization TGFPE emulsion

The rheological properties of PNF nanogel dispersion and TGFPE emulsion, including viscosity and visco-elasticity, were measured using a rheometer (Kinexus Ultra+; Malvern Instruments Ltd.) with a parallel plate (PP50, Φ = 50 mm; the gap was set at 0.5 mm). Conventional shear viscosity detection: Parameters were set as follows: temperature was 25°C, time of equilibrium temperature was 5 min, shear rate

was 300 S^{-1}, and detection duration was 2 min. Shear viscosity detection at different shear rates: The parameters were set as follows: the temperature was 25°C, the equilibrium temperature time was 5 min, the shear rate varied from 0 to 1000 s^{-1}, and the number of detection points was 50. Modulus detection at different temperatures: Parameters are set as follows: equilibrium temperature time is 5 min, temperature range is 25-45°C, heating rate is 2 ^{o}C/min, frequency is 1 Hz, shear stress is 0.5 Pa.

Yield stress detection: Parameters are set as follows: temperature is 37°C, equilibrium temperature time is 5 min, shear stress is 0.01 Pa, 0.05 Pa, 0.1 Pa, 0.5 Pa, 1 Pa and so on, the detection time under each shear stress condition is 2 min until the yield stress of the sample is detected. Creep and creep recovery detection: The parameters are set as follows: the temperature is 37°C, the equilibrium temperature time is 5 min, the shear stress is 0.5 Pa, and the detection time is 4 min (creep and creep recovery process are 2 min each). The zero-shear viscosity of the sample was calculated according to the following formula.

$$\eta_0 = 1/\tan\alpha \qquad \text{Formulation 8}$$

Where α is the inclination Angle of the linear fitting equation of the curve in creep stage.

***In vitro* ultrasonic imaging and X-ray imaging characterization of TGFPE emulsion**

Gel carriers were made first. Then, 1.5 mL sample (TGFPE emulsion or PNF10 nanogel dispersion) was injected into 1 mL dropper head, which was immersed in 37°C water bath at a constant temperature for 5 min. After TGFPE emulsion and

PNF10 nanogel dispersion gelled, the dropper head was quickly inserted into the gel carrier sample tank, and the temperature of the gel carrier and sample was maintained at 37°C for the whole process through 37°C water bath. The samples were characterized by ultrasonic imaging (GE Healthcare, Princeton, NJ, USA) at different points of time after gelation. Ultrasonic imaging parameters were set as follows: the mechanical index was 1.5, the frequency was 8 MHz and the gain was 0 dB.

X-ray attenuation evaluation was performed using computed tomography (CT) scanner (SOMATOM Sensation 64 Spiral; Siemens) at 100 kV and 120 mA in body PCT mode. 100 μL of PNF dispersions and various concentrations of Omnipaque (iodine concentration, 320 mg/mL) were added to a 96-well plate, and the entire region of each well was selected as the region of interest (ROI) for the sample in the well. The gray value of the ROI was measured using the built-in CT software for X-ray attenuation evaluation.

Renal embolization experiment

The process of vascular embolization operation

Animals were divided into groups of TGFPE emulsion, PNF10 nanogel dispersion, lipiodol and NS, with four rabbits in each group. The experimental rabbits were anesthetized by injection of chloral hydrate, and was fixed on the board. After that, their right hind limb groins were depilated and disinfected. The right femoral artery of each rabbit was isolated and the distal femoral artery was ligated by surgical sutures. After lifting the reserved silk thread near the proximal end, the femoral artery was punctured with an 18 G surgical puncture needle. The guide wire was introduced

after the withdrawal of the needle core, and the outer cannula of the puncture needle was withdrawn. Then the 4 F (Terumo, Tokyo, Japan) was introduced and fixed with the previously reserved silk thread. Iohexol contrast agent (Omnipaque, 300 mg I/mL, 0.5 mL/s) was injected into the femoral artery of the right leg of the experimental rabbit through catheter, and X-ray imaging was carried out. The reference guiding map was set up for use. 4 F Cobra catheter was directed to the main renal artery under the guidance of digital subtraction angiography (DSA; Siemens BICOR T.O.P., Germany). Around 1.5 mL of the sample (TGFPE emulsion, PNF10 nanogel dispersion and lipiodol) and NS were injected into the renal vessels through the catheter. After the "smoke" phenomenon of the kidney was observed on the display screen, it was proved that the sample had been filled the renal vessels. 5 min after that, Iohexol contrast agent was injected through the catheter and DSA angiography was applied simultaneously to verify whether the embolic material successfully embolized the vessels.

Review after vascular embolization

Within 1 day after vascular embolization, kidneys embolized by fluorescently-labeled embolic materials (PNF10 nanogel dispersion was labelled by RhB (excitation wavelength is 555 nm and emission wavelength is 580 nm), TGFPE emulsion was labelled by RhB and coumarin 6 (excitation wavelength is 466 nm and emission wavelength is 504 nm), and lipiodol was labelled by coumarin 6) were isolated and their slices were observed by fluorescence scanning. Before and after sample injection, Iohexol contrast agent was injected into vessel through the catheter, and the whole

process of vascular embolization was recorded by DSA angiography. Ultrasonographic review of renal after being embolized was carried out at 0 d, 1 d, 3 d, 1 w, 2 w and 4 w under B-mode ultrasonography and color doppler ultrasonography respectively. The ultrasonic frequency in B-mode and color doppler mode is 8 MHz and 5.3 MHz, respectively. Image J was used to calculate the gray value of ultrasonic-imaging enhanced region in the results of B-mode ultrasonography. CT scanning review (Somatom Definition AS 128 Siemens, Erlangen, Germany) was performed at 1 w, 2 w and 4 w after renal embolization in experimental rabbits.

VX2 liver cancer experimental rabbit TAE treatment model

The process of tumor embolization

The VX2 tumor model in adult New Zealand white rabbits (weight: 2.5–3.0 kg) were prepared as previously reported [1, 2]. Briefly, the rabbits were anesthetised intra-venously with sodium pentobarbital (2.0 wt.%, 30 mg/kg). The livers of the rabbits were then exposed, and the resulting VX2 tumor particles that are 1.0 mm^3 in size were transplanted into the left liver lobe. VX2-tumor-bearing rabbits were successfully obtained 17 days after tumor implantation. In order to evaluate the TAE therapy of TGFPE emulsion on liver tumors, 24 VX2-tumor-bearing rabbits were randomly divided into four groups (6 rabbits per group). The embolic material was injected at a rate of 0.5 mL/s. Lipiodol/Gelfoam, TGFPE emulsion, PNF10 nanogel dispersion and normal saline (*ca.* 0.3 mL per rabbit) were injected without any dilution because of their good flowability based on angiography.

review after embolization

DSA imaging: Before and after sample injection, iohexol contrast agent was injected into vessel through catheter for DSA imaging; DSA imaging was used to record the whole process of vascular embolization of tumor.

Evaluation of ultrasonic imaging: Ultrasonographic review of tumor was performed in experimental rabbits at 1 d before embolization, 1 d, 3 d, 1 w and 2 w after embolization under B-mode ultrasonography and color ddoppler ultrasonography, respectively. The ultrasonic frequency in B-mode and color doppler mode is 8 MHz and 5.3 MHz, respectively. Image J was used to calculate the gray value of ultrasonic-imaging enhanced region in the results of B-mode ultrasonography.

(3) CT scanning review: CT (Somatom Definition AS 128 Siemens, Erlangen, Germany) scanning review was performed before embolization, 1 w and 2 w after embolization. Tumor size was calculated after the reconstruction of CT scanning image, and tumor growth rate at different time points was calculated according to the following formula:

$$V = A \times B^2 \times \pi/6 \qquad \text{Formulation 9}$$

$$\text{Tumor Growth Rate}(\%) = \frac{(V_t - V_0)}{V_0} \times 100 \qquad \text{Formulation 10}$$

Where A is the maximum diameter of the maximum section of the tumor, B is the transverse diameter, V_0 and V_t are the tumor volumes of 1 w and 2 W before and after TAE, respectively.

The tumor necrosis rate (TNR) was calculated as the area percentage of the necrotic region in the total region of the tumor from the hematoxylin and eosin (H&E) stained sections, using the following equation:

$$TNR = [\frac{n}{n+t}] \times 100, \qquad \text{Formulation 11}$$

where, *n* denoted the necrotic area and *t* denoted the tumor cell area [3, 4].

TGFPE emulsion was injected into the kidneys of normal experimental rabbits under the guidance of ultrasound imaging technology

The catheter was superselected to the renal artery, The ultrasonic probe was adjusted to align with the kidney of the experimental rabbit, and then TGFPE emulsion and normal saline were injected into the blood vessels of the kidney part through the catheter under the guidance of ultrasonic imaging, respectively. The dynamic process of the infusion was observed and recorded.

Biocompatibility evaluation

Cytotoxicity test

HUVEC cells were used for evaluating the cytotoxicity of TGFPE emulsion. Firstly, the cells stored in the liquid nitrogen were resuscitated and cultured in 1640 complete medium for getting enough cells. Then, cells cultured in the culture bottle were digested from bottle by trypsin and diluted with complete medium to a suitable concentration for later use. The above cells suspension was using for seeding cells into the 96-well plate, assuring that there were 6000 to 8000 cells per well. And the cells would be incubated in an incubator for 1 day. TGFPE emulsion and PNF10 nanogel dispersion were diluted to different concentrations by using 1640 medium without serum. After removing the old medium, 100 μL samples with different concentrations were added into each well of the 96-well plate. The 96-well plate was placed in incubator for further culture for 1 day. After removing the original medium,

100 μL thiazolyl blue solution diluted with serum-free 1640 medium was added into each well, and the 96-well plate was put into an incubator for further culture for 4 h. 200 μL DMSO was added into each well after removing the old medium and the 96-well plate was stirred horizontally. The absorbance of each well was measured at the wavelength of 570 nm. Cell viability was calculated by the following formula.

$$\text{Cell viability (\%)} = {(T-B)}/{(C-B)} \times 100 \qquad \text{Formulation 12}$$

Where T, C and B are the absorbance of the experimental group (TGFPE emulsion and PNF10), the control group (without drug administration) and the blank group (including only medium), respectively.

Hemolysis test

The hemolysis evaluation was performed on the New Zealand rabbits as described in a previous report [5, 6]. 2 mL whole blood of normal experimental rabbits was taken and placed in a 10 mL EP tube coated with heparin sodium, and the tube was shaken to prevent coagulation of blood. Then 2 mL normal saline was added into the tube. After shaking, the tube was centrifuged at 1500 rpm for 10 min. After the above supernatant in the tube was removed, proper amount of normal saline was added into the tube. After that, the tube was centrifuged again at 1500 rpm for 25 min. Then discard the supernatant and repeat the above operation 3 times. The erythrocyte was suspended with normal saline to obtain 2% erythrocyte suspension.

TGFPE emulsion and PNF10 nanogel dispersion were diluted with normal saline to obtain sample diluents with different concentrations as the experimental group, normal saline as the group of negative control, and ultra-pure water as the group of

positive control. 150 μL samples of each group were added into 1.5 mL EP tubes, respectively, and kept warm in 37 °C water bath for 15 min. 150 μL 2% red blood cell suspension was added into the above EP tubes, and kept warm in 37°C water bath for 1 h. After that, tubes were centrifuged at 3000 rpm for 25 min and the supernatants were taken out and added into 96-well plate. Three parallel wells were set for all groups, and the absorbance value of samples in each group under the wavelength of 540 nm was detected by a microplate reader (1420 multilabel counter, Perkin-Elmer, MA, USA). The hemolysis rate of TGFPE emulsion and PNF10 nanogel was calculated according to the following formula:

$$\text{Hemolytic rate}(\%) = \frac{Abs_t - Abs_n}{Abs_p - Abs_n} \times 100 \qquad \text{Formulation 13}$$

Where Abs_t, Abs_n, and Abs_p are the absorbance values of the experimental group, negative control group, and positive control group, respectively.

Detection of liver and kidney function indicators

A biochemical auto-analyzer was employed to measure the liver and renal functions (Model DXC 8000; Beckman Coulter Diagnostics, Brea, CA, USA). Peripheral blood (1.0 mL) was collected on day 7 (i.e., prior to the therapy) and days 14 after the TAE therapy. The levels of plasma alanine aminotransferase (ALT), aspartate aminotransferase (AST), blood urea nitrogen (BUN), and serum creatinine (CRE) were measured.

Evaluation of histopathology and immunofluorescence

VX 2 tumor rabbits were sacrificed after TAE treatment for 2 weeks. Their heart, liver, spleen, lung and kidney were removed. Tissue slices of all the above organs were

observed under optical microscope after H&E staining. Histopathological analyses, such as H&E staining, terminal deoxynucleotidyl transferase dUTP nick-end labelling (TUNEL) and immunohistochemical staining, were further used to investigate the anti-tumor effect of TAE treatment. After TAE therapy on VX2-tumor-bearing rabbits or normal rabbits, these rabbits were humanely killed to collect various samples, including tumor tissues, peri-tumoral liver tissues and kidney tissues at various time intervals. All tissue samples were fixed in 10% buffered formalin, embedded in paraffin and cut into slices. The slides were de-waxed in xylene, hydrated using graded ethanol and stained for routine histologic evaluation by H&E. The sections were then observed microscopically (Leica DM3000) [7].

The TUNEL assay was used to stain and quantify apoptotic cells in the slices of tissue samples. According to the manufacturer's protocol, briefly, the slices were incubated with proteinase K (20 μg/mL) at 37 °C for 20 min and then were stained with TUNEL (1 : 9; Roche Diagnostics GmbH, Mannheim, Germany) at 37°C for 1 h in the dark after being rinsed with pure water. Afterwards, the slices were further stained for 5 min with 4′,6-diamidino-2-phenylindole dihydrochloride (DAPI). CLSM (Eclipse Ti; Nikon Corporation, Tokyo, Japan) was used to examine tumor cell apoptosis in various slices.

Before immunohistochemical analyses, the slices were immersed in a citrate buffer (0.01 mmol/L, pH = 6.0) and heated three times in a microwave oven (5 min each time). Endogenous peroxidase was blocked with methanol containing 3% hydrogen peroxide for 10 min. In order to implement various immunofluorescent

analyses, the slices were incubated overnight at 4°C with a 150-fold diluent of mouse anti-Ki-67 antigen (Dako, Carpinteria, CA, USA), a 100-fold diluent of mouse anti-HIF-1α (Thermo, IL, USA), a 150-fold diluent of mouse anti-VEGF (Millipore, Billerica, MA, USA) and a 50-fold diluent of mouse anti-human CD31 (Dako, Copenhagen, Denmark) monoclonal antibodies, respectively. Finally, these slices were incubated at 37°C in sequence with a 150-fold diluent of goat anti-rabbit secondary antibody (Aspen, Wuhan, China) for 50 min and a 200-fold diluent of fluorescent secondary antibody fluorescein isothiocyanate (FITC) (1 : 200, Wuhan Servicebio Biological Technology Co., Ltd., Wuhan, China) or Cyanine 3 (Cy3) (1 : 50, Wuhan Servicebio Biological Technology Co., Ltd.) for 60 min after being washed with a PBS solution three times for 5 min. Lastly, the slices were stained with DAPI to label the nuclei of tumor cells. CLSM (Eclipse Ti; Nikon Corporation, Tokyo, Japan) was used to investigate the expression levels of HIF-1α, VEGF and CD31 (Cy3, Ex/Em = 554 nm/650 nm; FITC, Ex/Em = 488 nm/525 nm; DAPI, Ex/Em = 350 nm/460 nm).

Image-Pro Plus 6.0 software (Media Cybernetics, Warrendale, PA, USA) is an image analysis software package that is used to quantify the average fluorescent intensity of five regions of tissue images (magnification: 400×). Apoptosis, proliferation and HIF-1α were assessed as follows: Single cells were scored as positive when a fluorescence signal for a specific marker was detected, and values are expressed as a percentage of positive cells with respect to the total number of nuclei (% mean of positive cells with respect to the mean total number of nuclei). VEGF was

defined as the integrated optical density (IOD). Five photographs per specimens were recorded at 400 fold magnification, to measure IOD of VEGF expressions in each group at different time points. Photographs were taken from the border region of the tumor in the control and TAE groups and the residual tumors surrounding coagulated tissue. Image analysis was done by the Image-Pro Plus software (Media Cybernetics, Warrendale, Pennsylvania). The IOD of VEGF expression was calculated automatically. Microvessel density (MVD) was computed by using the manual counting method. The sections were first scanned at 400× magnification to locate the areas of the most intense vascularization, and then MVD was counted in the fields of 400× magnification. MVD was represented as the mean value of the counts of the six fields.

References

1. Li L, Zhang HS, Zhao H, Shi DW, Zheng CS, Zhao YB, Yang XL. Radiofrequency-thermal effect of cisplatin-crosslinked nanogels for triple therapies of ablation-chemo-embolization. Chem Een J. 2022; 450: 138421.

2. Li H, Qian K, Zhang H, Li L, Zheng C, Zhao Y, Yang X. Pickering gel emulsion of lipiodol stabilized by hairy nanogels for intra-artery embolization antitumor therapy. Chem Een J. 2021; 418: 129534.

3. Fernandez M, Semela DJ, Bruix J, Colle I, Pinzani M, Bosch J. Angiogenesis in liver disease. J. Hepatol. 2009; 50: 604-620.

4. Sergio A, Cristofori C, Cardin R, Pivetta G, Ragazzi R, Baldan A, Girardi L, Cillo U, Burra P, Giacomin A, Farinati F. Transcatheter Arterial Chemoembolization (TACE)

in Hepatocellular Carcinoma (HCC): The Role of Angiogenesis and Invasiveness, Am. J. Gastroenterol. 2008; 103: 914-921.

5. Li L, Guo XP, Peng XL, Zhang HS, Liu YM, Li H, He XJ, Shi DW, Xiong B, Zhao YB, Zheng CS, Yang XL. Radiofrequency-responsive dual-valent gold nanoclusters for enhancing synergistic therapy of tumor ablation and artery embolization. Nano Today. 2020; 35: 100934.

6. Liu YM, Peng XL, Qian K, Ma YY, Wan JS, Li H, Zhang HS, Zhou GF, Xiong B, Zhao YB, Zheng CS, Yang XL. Temperature sensitive *p*(N-isopropylacrylamide-*co*-acrylic acid) modified gold nanoparticles for trans-arterial embolization and angiography. J. Mater. Chem. B. 2017; 5: 907-916.

7. Li L, Liu Y, Li H, Guo X, He X, Geng S, Zhao H, Peng X, Shi D, Xiong B, Zhou G, Zhao Y, Zheng C, Yang X. Rational design of temperature-sensitive blood-vessel-embolic nanogels for improving hypoxic tumor microenvironment after transcatheter arterial embolization. Theranostics. 2018; 8: 6291-6306.

Supporting Figures and Tables

Table S1 Synthesis formula of PNF nanogels

	PNF0	PNF10	PNF5	PNF2	PNF-RhB-10
NIPAM / mmol	30	30	30	30	30
MBA / mmol	1.5	1.5	1.5	1.5	1.5
SDS / mmol	0.66	0.66	0.66	0.66	0.66
KPS / mmol	0.4	0.4	0.4	0.4	0.4
H_2O / mL	340	340	340	340	340
HFBMA / mmol	0	3	6	15	3
AM / mmol	0	0	0	0	0.1
RhB / mmol	0	0	0	0	0.05
NIPAM / HFBMA	0	10	5	2	10

Table S2 The elemental analysis of PNF nanogels

	N（%）	C（%）	H（%）	S（%）
PNF0	11.5	56.74	9.12	0.123
PNF10	9.75	54.18	8.304	0.226
PNF5	8.5	52.61	7.651	0.146
PNF2	6.46	48.95	6.643	0.137

Table S3 The structural composition of PNF nanogels

PNF	PNF2	PNF5	PNF10
EA	2.8: 1	6.5: 1	16.5: 1
1H-NMR	2.9: 1	7.1: 1	16.7: 1

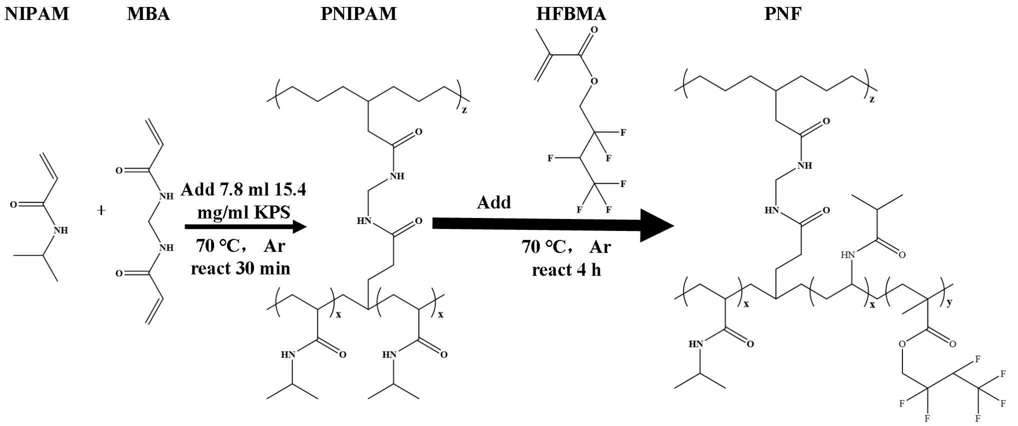

Fig. S1. The two-step feed emulsion polymerization of PNF nanogels.

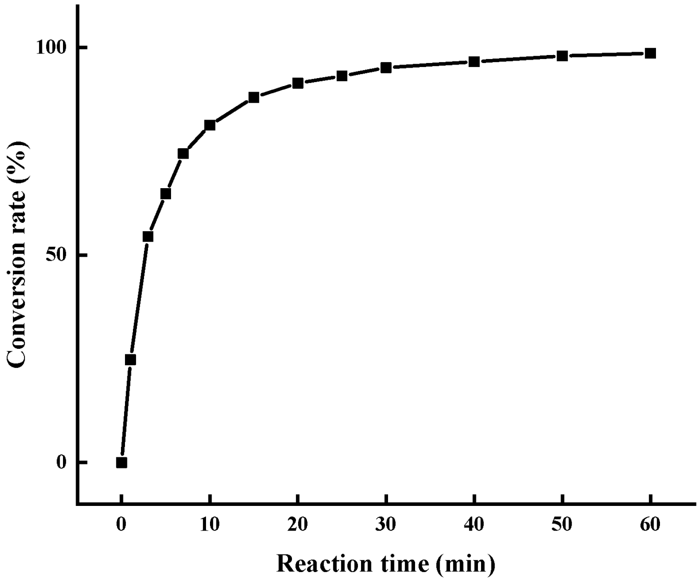

Fig. S2. Polymerization kinetics of PNIPAM core.

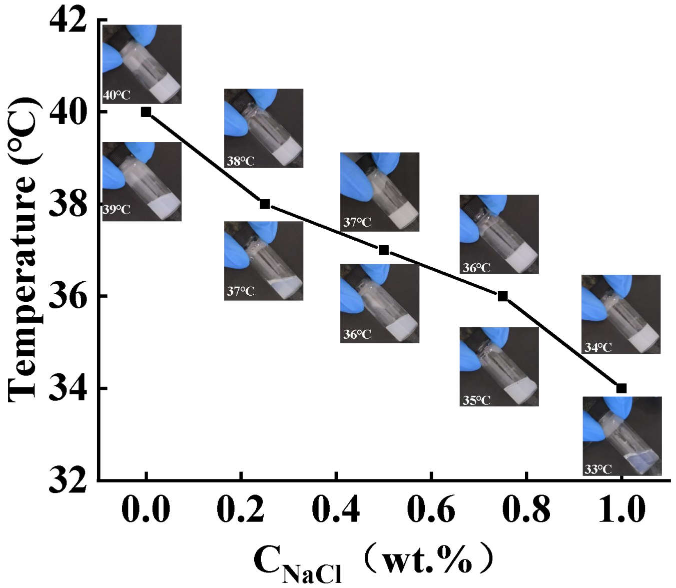

Fig. S3. NaCl concentration-temperature phase diagram of 5.5 wt.% PNF10 nanogel dispersion.

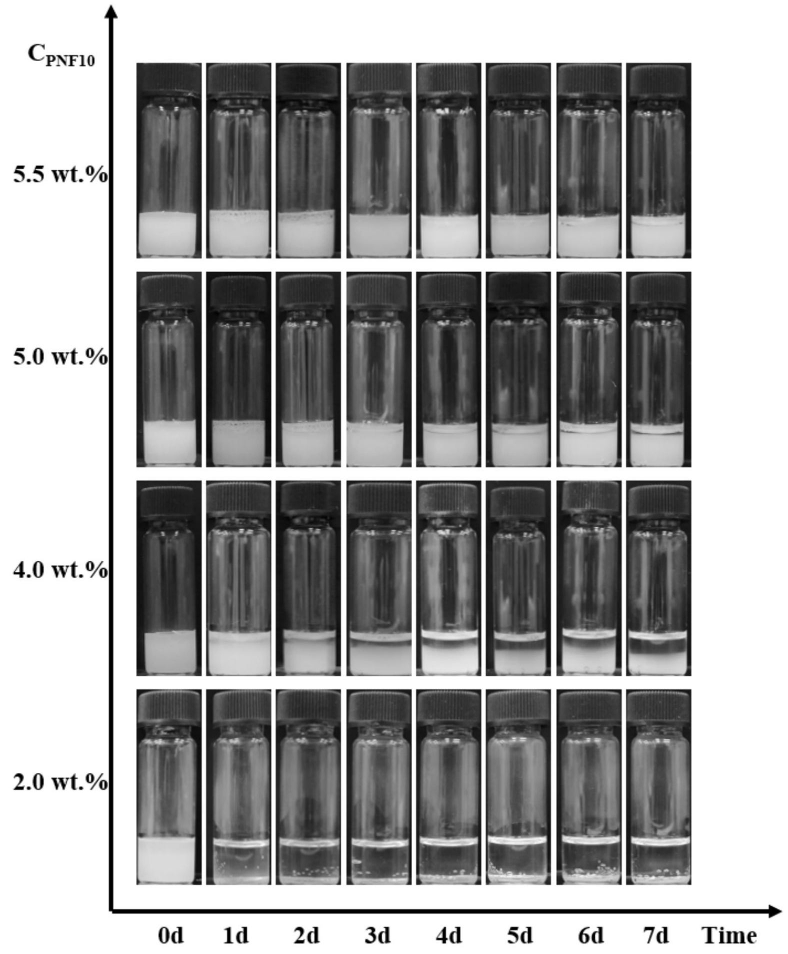

Fig. S4. Macroscopical observation of TGFPE emulsions stabilized by PNF10 nanogel dispersions with different concentration.

6 wt.% 7 wt.%

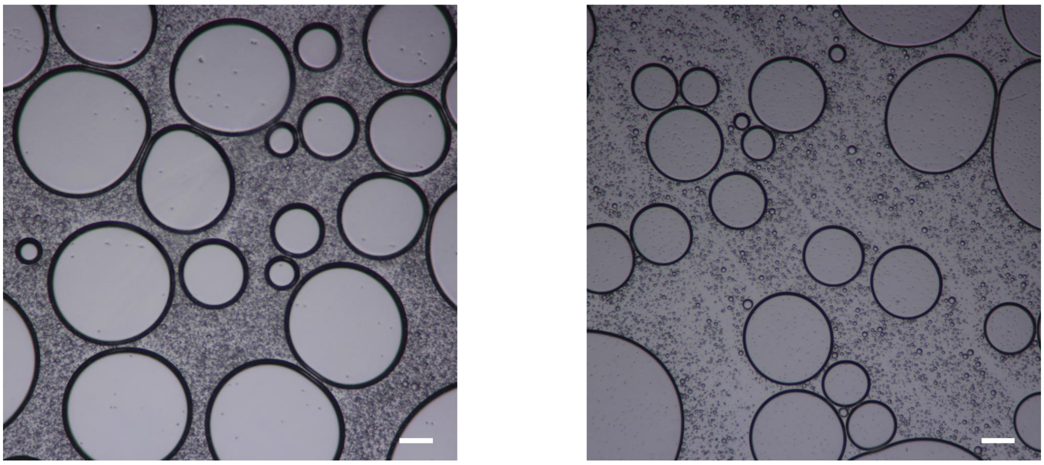

Fig. S5. Microscopic observation of TGFPE Emulsion stabilized by 6 wt.% and 7 wt.% PNF10 nanogel. Scale = 250 μm.

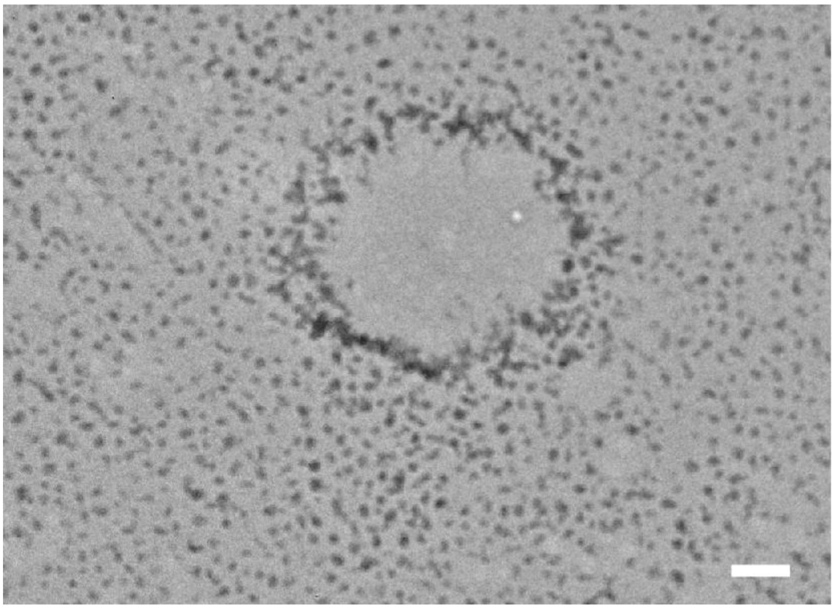

Fig. S6. TEM observation of TGFPE emulsion. Scale = 250 μm.

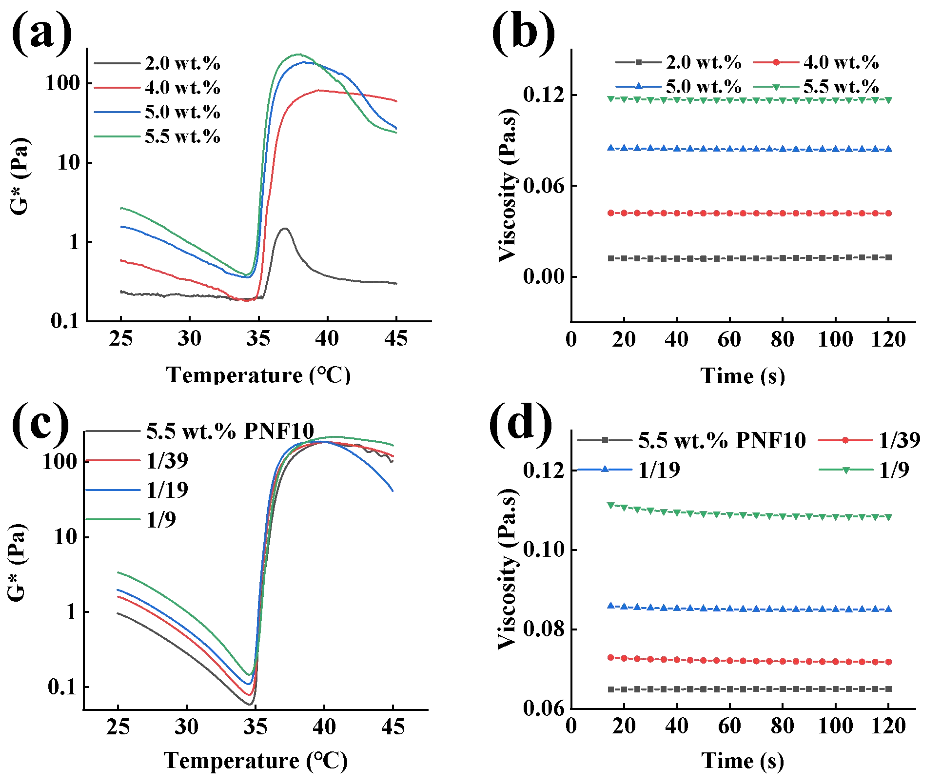

Fig. S7. Modulus and viscosity detection of TGFPE emulsions. **a)** and **b)** are modulus and viscosity detection of TGFPE emulsions stabilized by PNF10 nanogel dispersions with different concentration, respectively. **c)** and **d)** modulus and viscosity detection of TGFPE emulsions with different ratios of O/W.

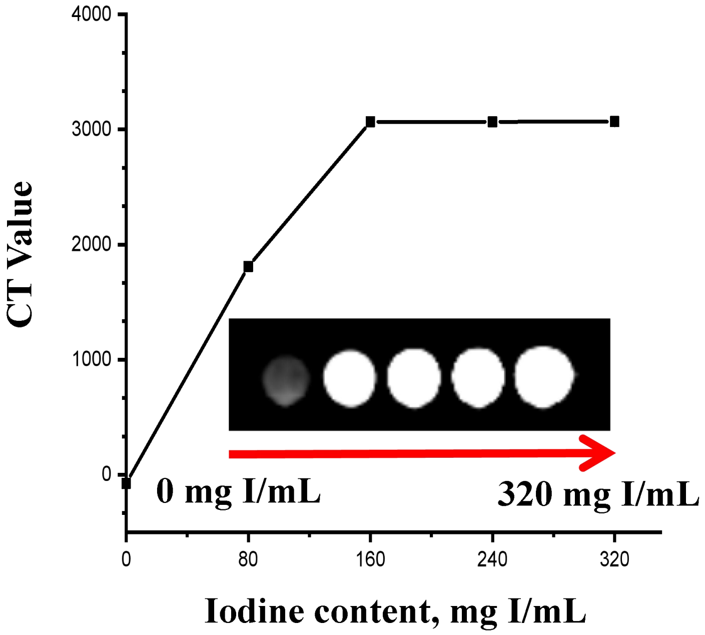

Fig. S8. CT value of TGFPE at virous iodine content. (0 mg I/mL, 80 mg I/mL, 160 mg I/mL, 240 mg I/mL, and 320 mg I/mL)

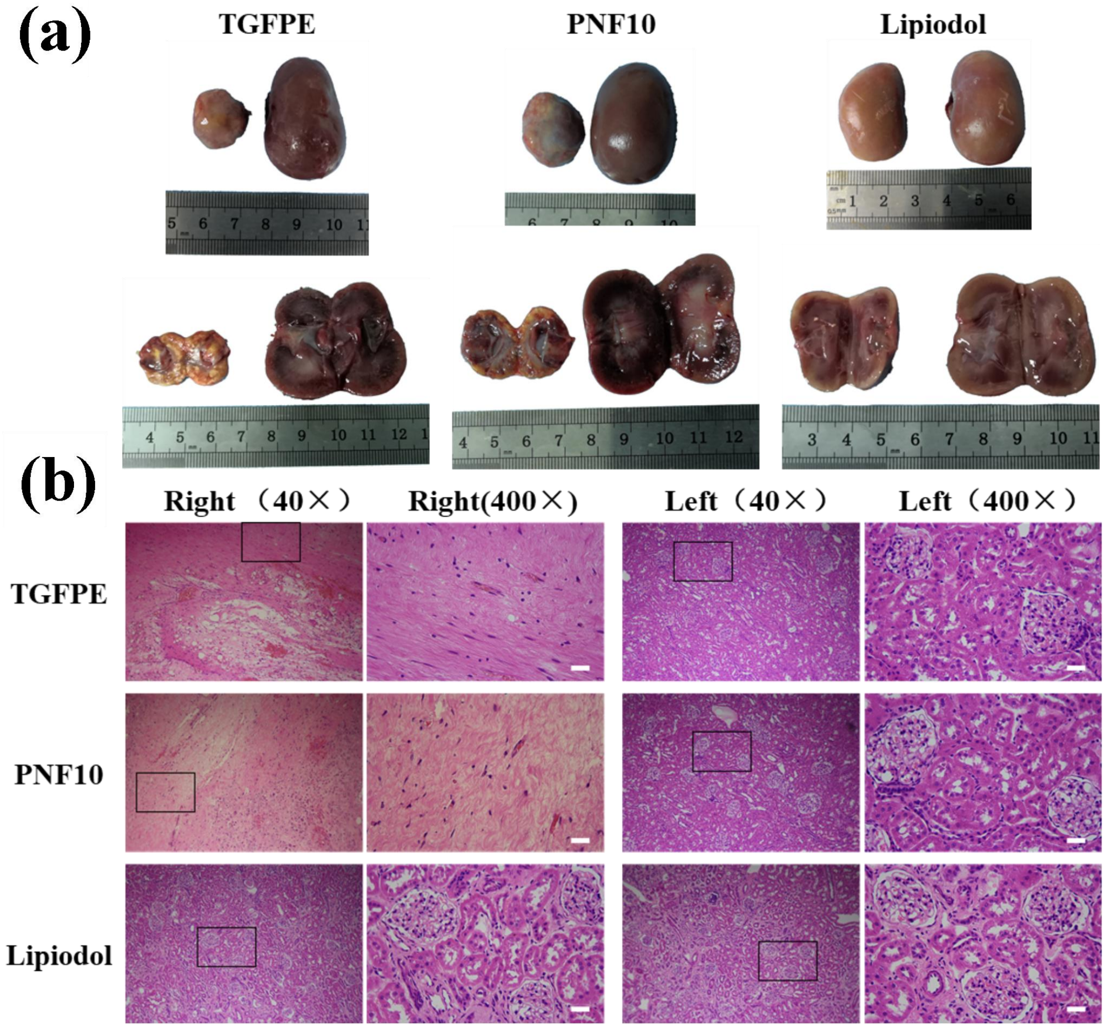

Fig. S9. Histopathological evaluation of rabbit kidney embolism with of TGFPE, PNF10 dispersion, lipiodol and NS. **a)** Photos of kidneys after vascular embolization with the above embolic materials for 4 weeks and **b)** the microscopic observation of kidney slices after H&E staining. Scale = 20 μm.

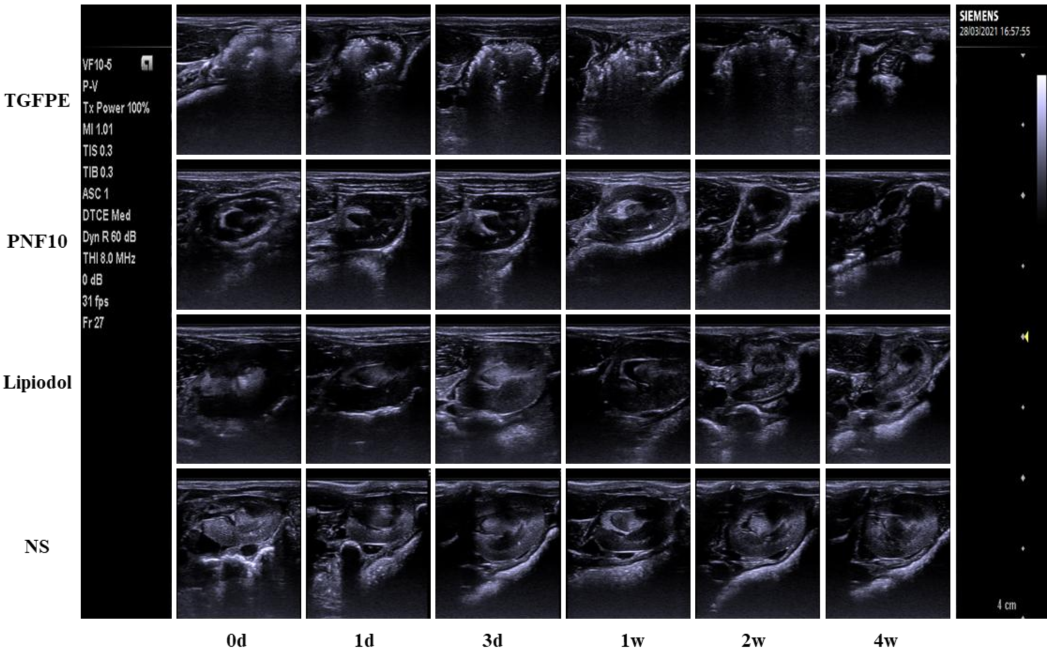

Fig. S10. B-mode ultrasound images of kidney after being embolized.

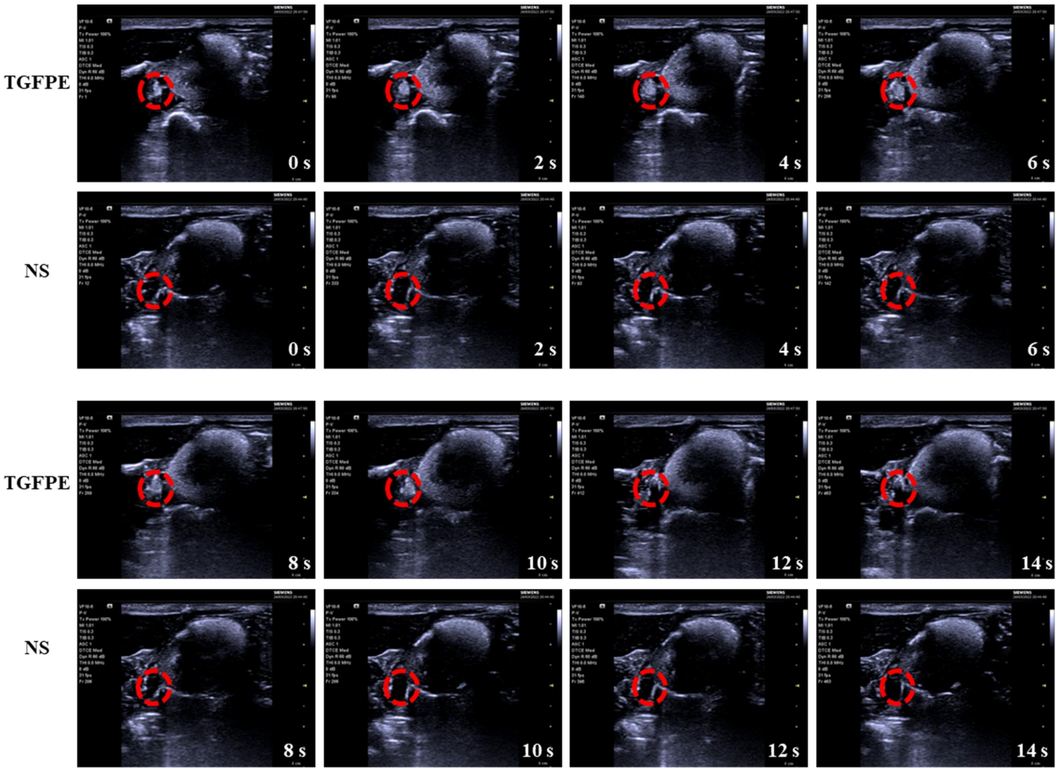

Fig. S11. The observation of the whole processes of injecting NS and TGFPE into the normal kidney under the guidance of ultrasound. The region surrounded by dotted red line is the renal aorta.

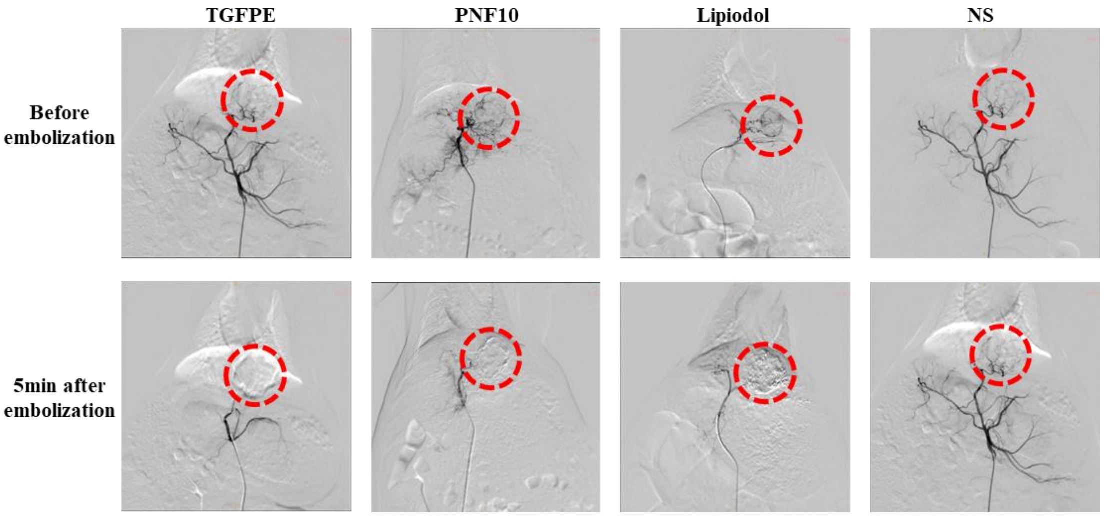

Fig. S12. DSA images of tumors before and after embolized for 5 minutes. The region surrounded by dotted red line is the embolized tumor.

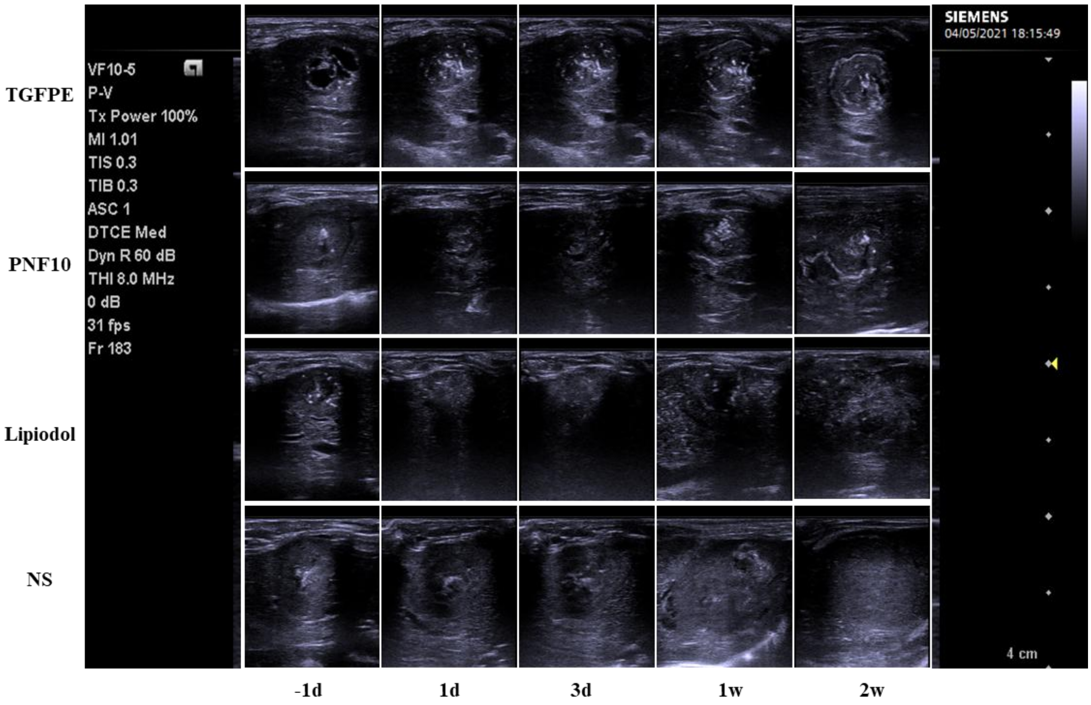

Fig. S13. B-mode ultrasound images of tumors after being embolized.

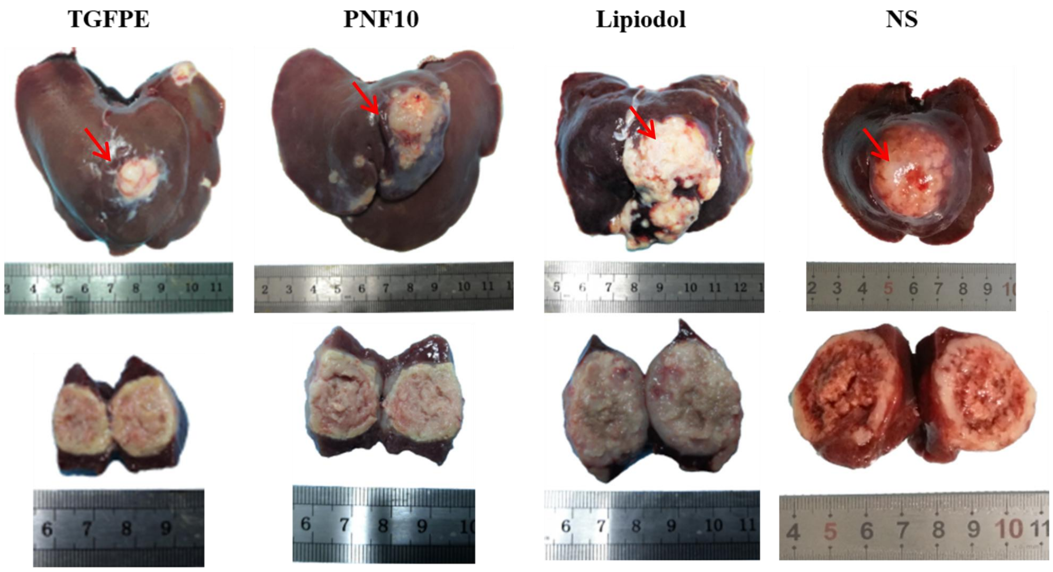

Fig. S14. The photos of liver and tumor after 14 days of embolization. The region where red arrow pointed at was tumor.

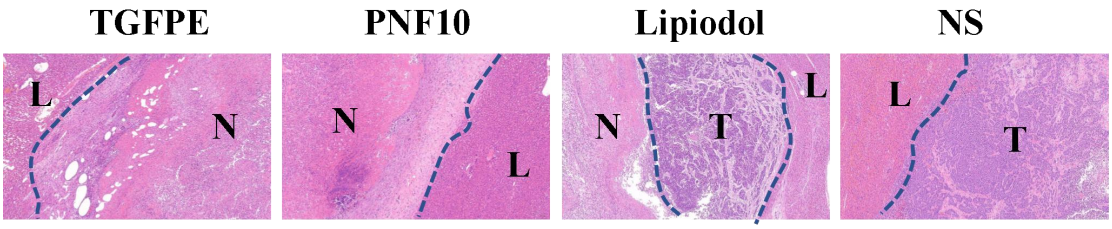

Fig. S15. H&E staining of different groups after embolization on day 14 (original magnification, × 100). (T) areas of tumor (L) liver tissue (N) areas of necrosis.

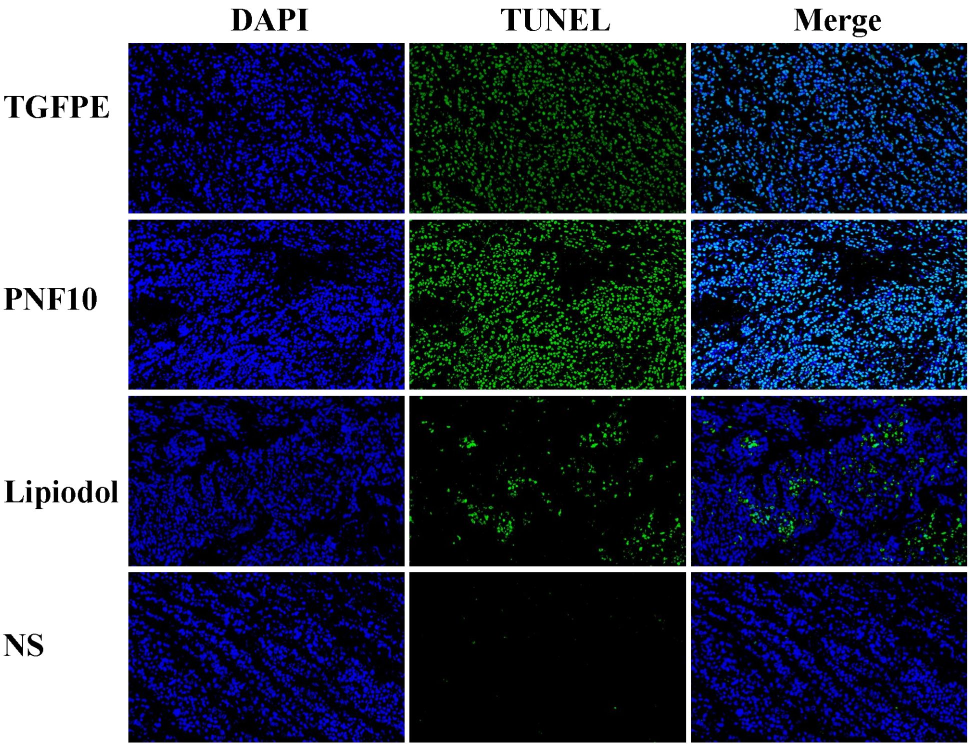

Fig. S16. Confocal fluorescence microscopy images of DAPI (blue color) and TUNEL (green color), (original magnification, ×400).

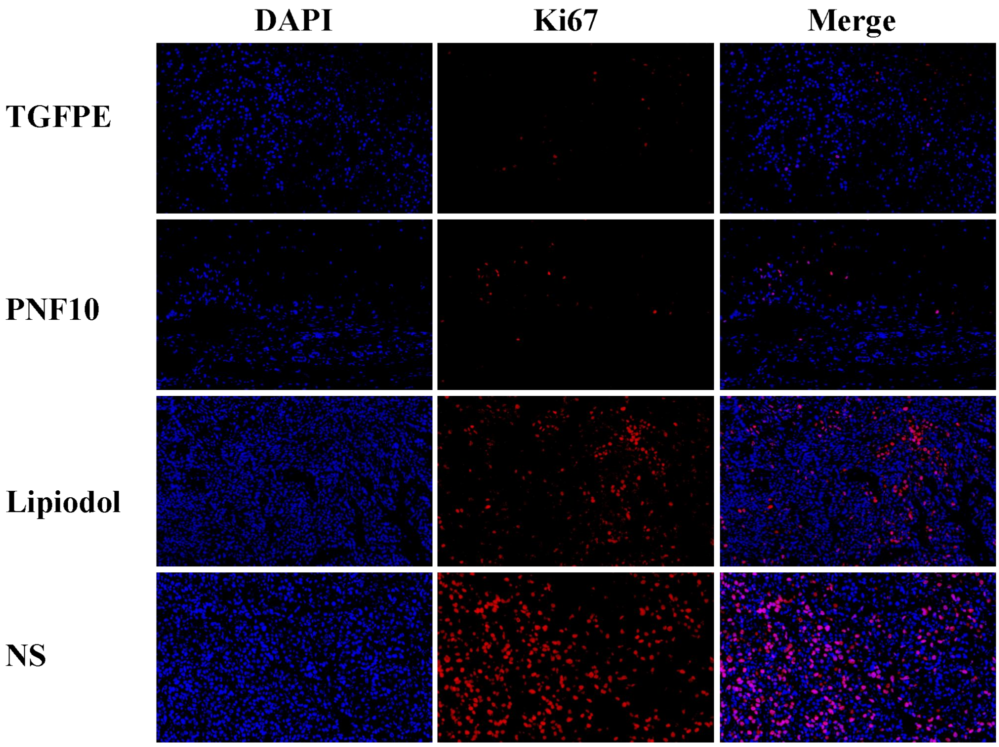

Fig. S17. Confocal fluorescence microscopy images of DAPI (blue color) and Ki67 (red color), (original magnification, ×400).

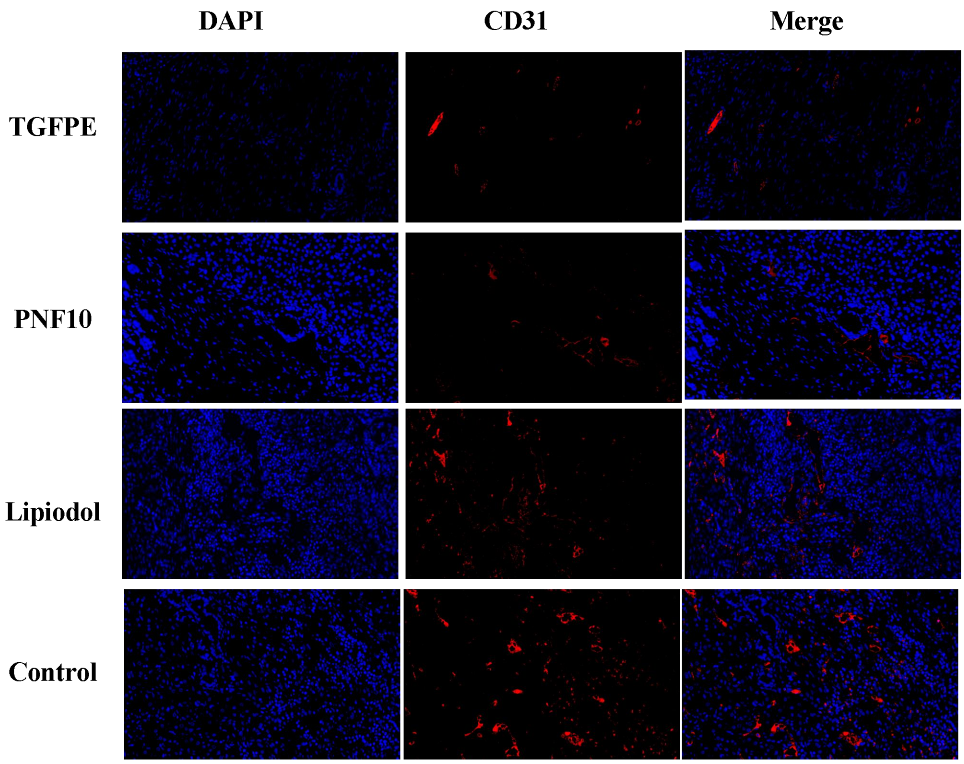

Fig. S18. Confocal fluorescence microscopy images of CD31 surrounding the residual tumors at different time points, (original magnification, ×400).
